# ADAM17 as a promising therapeutic target: from structural basis to inhibitor discovery in human diseases

**DOI:** 10.3389/fphar.2025.1640090

**Published:** 2025-09-19

**Authors:** Lisa Liu, Erkang Tian, Shuqi Quan, Chongying Su, Jiawei Zhou, Sijia Hu, Nanyan Bian, Shufang Du, Juan Li

**Affiliations:** State Key Laboratory of Oral Diseases, National Center for Stomatology, National Clinical Research Center for Oral Diseases, Department of Orthodontics, West China Hospital of Stomatology, Sichuan University, Chengdu, China

**Keywords:** ADAM17, therapeutic target, inflammation, cancer, cardiovascular diseases, neurological disorders, small-molecule inhibitors

## Abstract

A disintegrin and metalloproteinase 17 (ADAM17) is a transmembrane protease that regulates diverse physiological processes by shedding membrane-bound proteins, including cytokines, their receptors, and adhesion molecules. A mounting body of evidence has emerged linking ADAM17 to the pathogenesis of various diseases, including inflammation, cancer, cardiovascular and neurodegenerative diseases, highlighting its potential as a therapeutic target. This review offers a comprehensive overview of the molecular structure and biological functions of ADAM17, emphasizing its role in human diseases and therapeutic strategies that target ADAM17 activity. Recent advances in the development of ADAM17-targeting agents, including small-molecule inhibitors, monoclonal antibodies, and endogenous regulatory proteins, are discussed with a focus on the structural basis of their activity, with the aim of informing and guiding future drug discovery efforts.

## 1 Introduction

ADAM17, belonging to the A Disintegrin and Metalloproteinase (ADAM) family, was initially named tumor necrosis factor-alpha converting enzyme (TACE) because it was primarily considered to be the protease engaged in the cleavage of membrane-bound TNF-α (Black et al., 1997; Moss et al., 1997). However, subsequent studies have revealed that ADAM17 is responsible for the proteolytic shedding of more than 80 membrane-associated substrates, which can be broadly categorized into cytokines (e.g., TNF-α) and cytokine receptors (e.g., TNF receptors, IL-6R), ligands of growth factors receptors (e.g., amphiregulin), adhesion molecules (e.g., L-selectin, ICAM-1, VCAM-1), and other membrane proteins (e.g., amyloid precursor protein, fractalkine, neuregulins) (Düsterhöft et al., 2019a; Scheller et al., 2011). This broad substrate repertoire underpins ADAM17’s pleiotropic roles in regulating immune responses, inflammation, neurobiology, tissue homeostasis, and developmental signaling.

Dysregulation of ADAM17 activity has been implicated in various diseases, including inflammatory disorders, cancer cardiovascular and neurodegenerative diseases. Its ability to process tumor necrosis factor-alpha (TNF-α) and epidermal growth factor receptor (EGFR) ligands makes it a critical player in disease progression and a promising therapeutic target (Lisi et al., 2014). Over the past decades, substantial efforts have been made to develop ADAM17 inhibitors, ranging from small-molecule compounds to monoclonal antibodies, with the aim of modulating its activity for therapeutic benefit.

In this review, we provide a comprehensive overview of ADAM17, including its structural characteristics, biological functions, and pathological significance. Furthermore, we summarize the latest advancements in ADAM17 inhibitor development and discuss the potential directions for future research and therapeutic applications.

## 2 Structure of ADAM17

**FIGURE 1:**
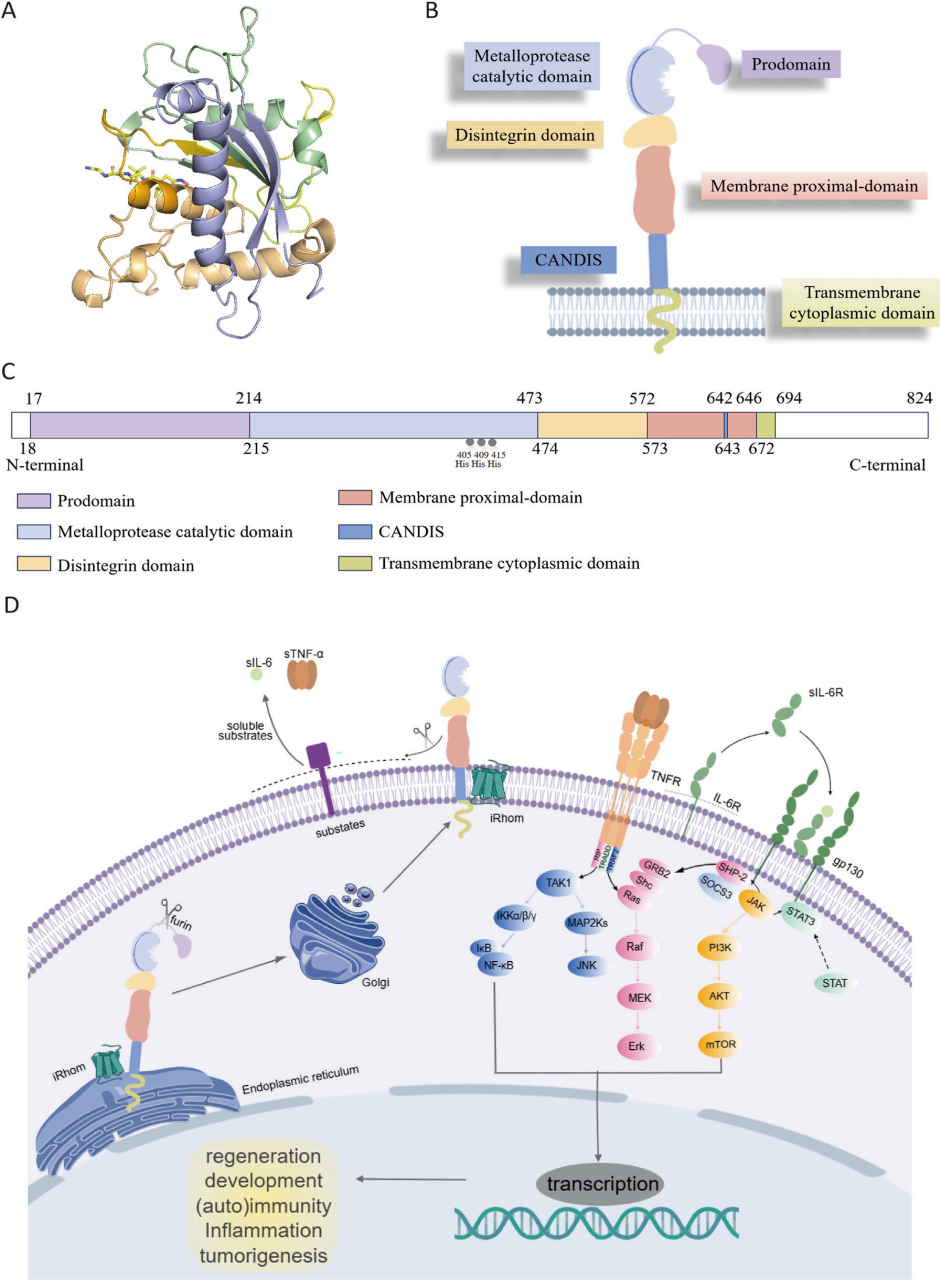
**(A)** Crystal structure of catalytic domain of ADAM17 (PDB ID: 1BKC). **(B)** Overview of ADAM17. **(C)** The primary structure of ADAM17. **(D)** iRhoms bind immature proADAM17 in the endoplasmic reticulum and transport it to the Golgi apparatus, where the inhibitory prodomain is proteolytically removed by furin-like proteases. Subsequently, mature ADAM17 is delivered to the cell membrane and cleave its substrates such as TNF-α and IL-6R, thereby participating in various cell signaling pathways. ADAM17 (disintegrin and metalloprotease 17); TNFR (tumor necrosis factor receptor); IL-6R (interleukin 6 receptor); gp130 (glycoprotein 130); STAT (signal transducerand activator of transcription); TAK1: transforming growth factor beta activated kinase 1; IKKα/β/γ(IκB kinase complex); IκB (inhibitor of NF-κB); NF-κB (nuclear factor kappa-light-chain-enhancer of activated B cells); MAP2Ks (mitogen-activated protein kinase kinases); JNK (c-jun N-terminal kinase); GRB2 (growth factor receptor-bound protein 2); SHC (src homology and collagen domain containing); Ras (rat sarcoma viral oncogene homolog); Raf (rapidly accelerated fibrosarcoma); MEK (mitogen-activated protein kinase); Erk (extracellular signal-regulated kinase); SHP-2 (src homology phosphatase 2); SOCS3 (suppressor of cytokine signaling 3); JAK (janus kinase); PI3K (phosphoinositol-3 kinase); AKT (protein kinase B); mTOR (mammalian target of rapamycin); STAT (signal transducer and activator of transcription).

ADAM17 is a type I transmembrane protein consisting of 824 amino acids, encoded by a gene located on chromosome 2p25 (Rose-John, 2013). The crystal structure of the ADAM17 metalloprotease domain (Figure 1A) exhibits the characteristic metzincin metalloprotease fold, comprising five α-helices and five distorted β-strands, which together form the functional scaffold for proteolytic activity (Maskos et al., 1998). The protein has distinct structural features and contains multiple domains: (1) N-terminal signal sequence (1–17 amino acids); (2) prodomain (PD, 18–214 amino acids); (3) metalloprotease catalytic domain (MD, 215–473 amino acids); (4) disintegrin domain (DD, 474–572 amino acids); (5) membrane proximal domain (MPD, 573–671 amino acids), containing a conserved ADAM17 interaction sequence (CANDIS, 643–646 amino acids); (6) transmembrane cytoplasmic domain (TMD, 672–694 amino acids); (7) C-terminal cytoplasmic domain (CD, 695–824 amino acids) (Figures 1B,C) (Chemaly et al., 2017). The N-terminal region interacts with the β1 and β3 sites, while the C-terminal region associates with the α5 site. It exists mainly in two forms, the full-length ADAM17 precursor protein (approximately 110 kDa) and the mature form lacking the prodomain (approximately 80 kDa) (Xu et al., 2016).

The N-terminal signal sequence directs the newly synthesized pro-ADAM17 (approximately 110 kDa) to the endoplasmic reticulum (ER) and then to the Golgi apparatus for further processing. The prodomain contains a shared cysteine switch sequence PKVCGY186 that connects the zinc ions in the catalytic center, maintaining ADAM17 n an inactive state during its transit from ER to the Golgi apparatus (Grötzinger et al., 2017). During activation, proprotein convertases such as furin are able to remove the prodomain, thereby generating the mature ADAM17 (Lopes et al., 2020). Additionally, the prodomain is essential for the proper folding and maturation of ADAM17. In its absence, the protein undergoes proteolytic degradation in the ER, indicating a protective function of the prodomain (Junior, 2020).

The MD functions as the main catalytic region of ADAM17, containing the conserved zinc-dependent HExGHxxGxxHD motif in which His405 and His409 directly coordinate the Zn^2+^ ion, while His415 participates via water-mediated coordination; Glu406 acts as a general base to activate the catalytic water molecule, and Asp418 stabilizes the zinc-binding geometry through a hydrogen-bond network (Saha et al., 2019). Tissue inhibitor of metalloproteinases-3 (TIMP3) can bind with high affinity to the catalytic center of ADAM17 and inhibit ADAM17 activity (Lee et al., 2002; Wisniewska et al., 2008). The DD is thought to be a scaffold for the extracellular structural domain of ADAM17, maintaining the rigidity of the C-type structure of the extracellular structural domain by connecting the catalytic structure to the near-membrane protein structural domains (Janes et al., 2005; Takeda et al., 2006). The cysteine-rich domain and epidermal growth factor (EGF)-like domain are integral components of the MPD, playing crucial roles in substrate recognition and protein shedding (Schmidt-Arras and Rose-John, 2019; Düsterhöft et al., 2019b). Additionally, the positively charged motif (Arg625-Lys628) of the MPD interacts with phosphatidylserine in the outer membrane, thereby modulating ADAM17 conformation and facilitating its activation (Calligaris et al., 2021). CANDIS is a highly conserved sequence positioned between MPD and TMD, forming an amphipathic helix that interacts with cell membranes (Düsterhöft et al., 2015; Imoto et al., 2021), which binds to the type I transmembrane protein IL-6R but not the type II transmembrane protein TNF-α (Düsterhöft et al., 2014). It plays a significant role in the function of ADAM17, particularly in substrate recognition and enzyme activity regulation. For example, mutations in specific amino acid residues within CANDIS, such as Asp647, can lead to functional abnormalities and affect its normal cellular function. The TMD and CD play essential roles in modulating the cellular response to signaling events associated with the extracellular domain. These domains facilitate the integration of extracellular signals into intracellular pathways, thereby influencing processes such as inflammation, cell proliferation, and tissue repair. Specifically, the TMD domain anchors ADAM17 to the cell membrane, ensuring its proper localization and interaction with membrane-associated signaling molecules. Meanwhile, the CD domain serves as a key interface for downstream signaling, allowing ADAM17 to transduce extracellular signals into intracellular responses through interactions with various adaptor proteins and signaling cascades (Lora et al., 2021).

## 3 Biological function of ADAM17

**FIGURE 2:**
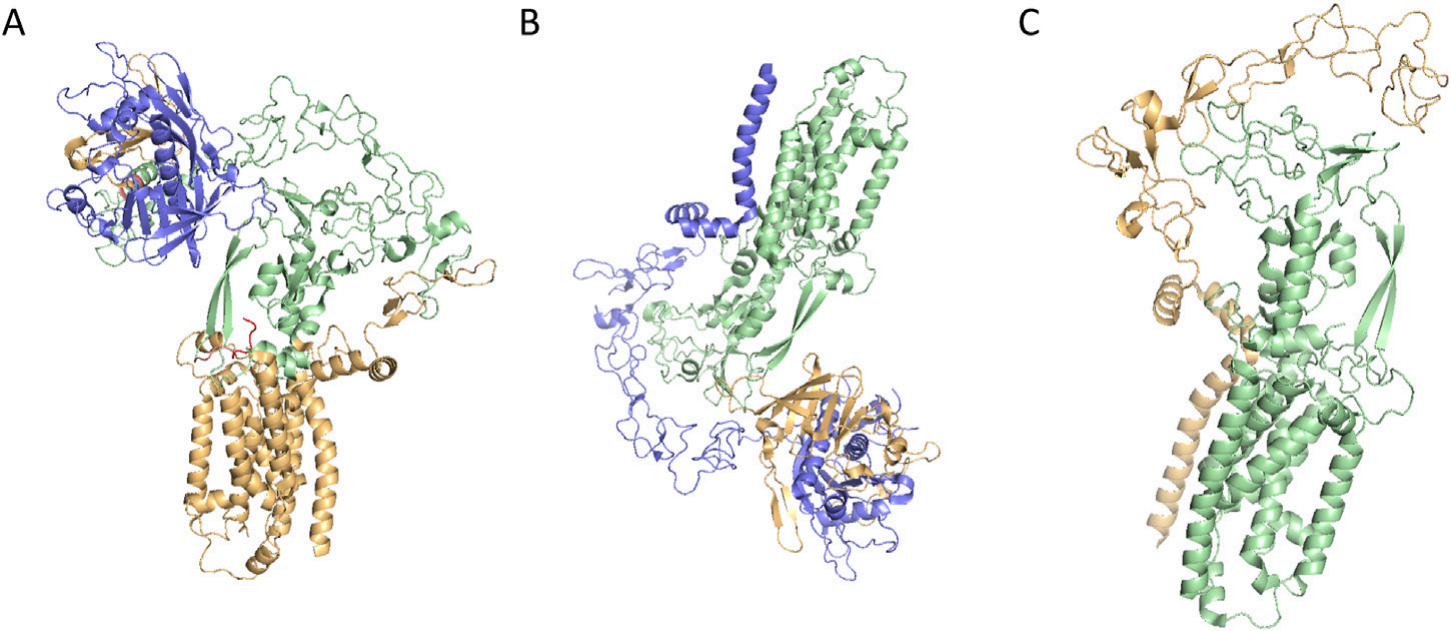
**(A)** Cryo-EM structure of ADAM17/iRhom2(PDB ID: 8SNL); **(B)** Cryo-EM structure of prodomain/mature ADAM17/iRhom2(PDB CODE: 8SNM); **(C)** Cryo-EM structure of mature ADAM17/iRhom2 (5 aa linker fusion) (PDB CODE: 8SNN).

ADAM17 is produced by a multitude of cell types in an inactive form featured a cysteine switch motif. Initially, iRhoms interact with immature proADAM17 in the ER, facilitating its transport to the Golgi apparatus, where furin-like proteases cleave the inhibitory prodomain. Subsequently, the mature ADAM17 is delivered to the cell membrane and cleave substrates such as TNF-α and IL-6R, thereby participating in various physiological and pathological processes including development, tissue repair, immunity, inflammation or tumor formation (Figure 1D) (Grötzinger et al., 2017). The function of ADAM17 is modulated through phosphorylation of its cytoplasmic tail by intracellular kinases, including protein kinase C (PKC), Polo-like kinase 2 (PLK2), and mitogen-activated protein kinase (MAPK). ADAM17 activation, as its surface exposure is closely associated with substrate shedding induced by various activators (Sommer et al., 2016). IRhom2, a pseudoprotease, plays a crucial role in regulating ADAM17 activity by retaining the cleaved prodomain, preventing premature activation and ensureing precise control over substrate shedding. Specifically, iRhom2 interacts with the TMD, facilitating its transport and maturation, while its unique extracellular domain unexpectedly retains the cleaved prodomain. This mechanism is essential for preventing untimely activation of ADAM17, thereby modulating the release of critical signaling molecules such as TNF-α and IL-6. Without iRhoms, ADAM17 remains trapped in the ER, preventing its maturation and its transport to the plasma membrane (McIlwain et al., 2012). Researchers have uncovered that the mature form of ADAM17 exhibits high dynamics, which is crucial for its ability to interact with substrates. The intricate relationship between iRhom2 and ADAM17 provides a foundation for understanding how this regulatory axis modulates critical signaling pathways and offers potential targets for diseases where ADAM17 activity is dysregulated (Figures 2A–C) (Lu et al., 2024).

ADAM17 is the major protease responsible for the proteolytic cleavage of several key substrates involved in immune and inflammatory responses, including TNF-α, the IL-6R, and ligands of EGFR (Schumacher and Rose-John, 2019). Specifically, ADAM17 cleaves TNF-α to generate its soluble form, which then binds to TNF receptors on the cell surface to mediate inflammatory signaling. Similarly, it cleaves the IL-6 receptor to produce soluble IL-6R, enhancing IL-6 signaling and promoting inflammation. Beyond immune regulation, it also contributes to cardiovascular disease-related pathways by mediating the cleavage of epidermal growth factor receptor (EGFR) ligands, which are implicated in the pathogenesis of conditions such as coronary artery disease, aneurysm, heart failure, and hypertension (Chemaly et al., 2017; Lu et al., 2011; Takayanagi et al., 2016). Furthermore, ADAM17 has been implicated in the modulation of the activity and migration of immune cells, including the shedding of T cell co-stimulatory and co-inhibitory receptors, thereby influencing T cell development and function (Sun et al., 2024). These functions highlight ADAM17’s central role in both physiological and pathological processes, making it a significant target for therapeutic interventions in diseases characterized by chronic inflammation and cancer. It is also involved in the regulation of the Notch signaling pathway, mediating cleavage of the Notch receptor under certain contexts. Following S1 cleavage of the Notch precursor by Furin in the Golgi, which generates a mature heterodimer at the cell surface, ligand binding (e.g., Delta/Jagged) induces a conformational change that exposes the S2 site for cleavage by ADAM10/ADAM17, producing a membrane-bound truncated intermediate. Subsequent γ-secretase–mediated S3 cleavage releases the Notch intracellular domain (NICD), which translocates to the nucleus to activate transcription of downstream genes, thereby exerting profound effects on cell fate determination and tissue development (Sen et al., 2023; Jia et al., 2019; Murthy et al., 2012a; Alabi et al., 2021). While ADAM10 cleaves Notch1 under physiological, ligand-dependent conditions, ADAM17 mainly cleaves Notch1 under ligand-independent conditions (Alabi et al., 2021; Bozkulak and Weinmaster, 2009). In the tumor microenvironment, ADAM17 promotes the occurrence, development, invasion, and metastasis of tumors by remodeling the extracellular matrix (ECM) and affecting the migration of immune cells. It can also regulate receptors on immune cells by cleaving ligands on the surface of tumor cells, thereby affecting tumor immune surveillance (Schumacher and Rose-John, 2019). The function of ADAM17 is not limited to the fields of tumor and immunity; it also plays a role in the development and maintenance of the nervous system. For instance, ADAM17 is involved in the cleavage of the neurotrophin receptor p75, modulating the nerve growth factor (NGF) signaling pathway, and thereby regulating the survival and death of neurons (Li et al., 2013).

In summary, ADAM17, characterized by its diverse substrate specificity and extensive biological effects, is crucial for maintaining physiological homeostasis and regulating pathological processes. Given that the aberrant activation of ADAM17 has been implicated in the onset and progression of various diseases, targeting ADAM17 emerges as a promising therapeutic strategy for treating these related conditions.

## 4 The role of ADAM17 in diseases

### 4.1 Inflammation

In infectious inflammation, such as in lipopolysaccharide (LPS)-induced endotoxemia and sepsis models, ADAM17 activity results in the enhanced shedding of TNF-α and IL-6R, both of which are critical mediators of the innate immune response (Rahn and Becker-Pauly, 2022; Yan et al., 2016). These findings underscore the enzyme’s essential role in initiating and amplifying inflammatory signaling during acute infections.

In osteoarthritis (OA), ADAM17 expression is upregulated in articular cartilage and correlates with disease severity. Functional studies show that ADAM17-deficient mice exhibit reduced cartilage degradation, suggesting a pathological role in OA. In primary chondrocyte cultures, ADAM17 activation promotes the release of soluble TNF-α and ligands of EGFR, further contributing to inflammation and cartilage degradation. Intra-articular administration of ADAM17 inhibitors has been reported to attenuate disease progression in experimental OA models, indicating its therapeutic potential in managing this debilitating condition (Kaneko et al., 2022).

In the context of autoimmune diseases, aberrant ADAM17 activity has been linked to elevated TNF-α levels and chronic inflammation in conditions such as rheumatoid arthritis (RA), systemic lupus erythematosus (SLE), psoriasis, and Crohn’s disease. Beyond inflammation, recent studies implicate ADAM17 in promoting epithelial–mesenchymal transition (EMT), a process associated with tissue fibrosis, autoimmunity and chronic inflammation. These insights highlight the enzyme as a promising therapeutic target for modulating immune dysregulation and fibrotic progression in autoimmune settings (Dwivedi et al., 2024; Sisto and Lisi, 2024).

During SARS-CoV-2 infection, ADAM17 contributes to COVID-19 pathogenesis by promoting the shedding of angiotensin-converting enzyme 2 (ACE2), thereby disrupting its protective signaling axis. This cleavage event, alongside the increased release of active TNF-α, aggravates lung inflammation and contributes to the cytokine storm and neutrophil hyperactivation seen in severe cases. Preclinical data suggest that pharmacological inhibition of ADAM17 reduces pulmonary inflammation, diminishes leukocyte infiltration, and improves the neutrophil-to-lymphocyte ratio—an established marker of COVID-19 severity (Haga et al., 2008; Zipeto et al., 2020).

### 4.2 Cancer

Since its discovery, ADAM17 has been increasingly recognized as a key regulator in the tumor microenvironment. It promotes oncogenesis through the shedding of membrane-bound precursors of growth factors and cytokines, leading to activation of signaling pathways such as Notch and EGFR, which promote tumor cell proliferation, migration, and invasion (Sinnathamby et al., 2011). Furthermore, ADAM17 suppresses CD8^+^ T cell-mediated antitumor immunity by cleaving CD122 (the IL-2/IL-15 receptor β-chain), thereby impairing cytotoxic T cell function. Pharmacological or genetic inhibition of ADAM17 has been shown to enhance CD8^+^ T cell responses and improve the efficacy of chimeric antigen receptor (CAR) T cell therapies in solid tumors (Sun et al., 2024).

#### 4.2.1 Colorectal and gastric cancer

ADAM17 is implicated in the pathogenesis of colorectal cancer (CRC), with its elevated expression correlating with tumor progression, including deeper invasion, lymph node metastasis, and distant metastasis (Chen et al., 2024). Similarly, it was reported that high ADAM17 expression in gastric cancer is significantly associated with aggressive progression and poor prognosis, and may serve as an independent prognostic biomarker (Zhang et al., 2012; Fang et al., 2017).

#### 4.2.2 Non-small cell lung cancer (NSCLC)

In NSCLC, ADAM17 overexpression promotes the release of soluble IL-6 receptor (sIL-6R), correlating with lower survival rate (Ni et al., 2013). It also mediates the activation of the Notch signaling pathway, which is crucial for cell survival and resistance to antitumor drugs in NSCLC. Inhibition of ADAM17 can enhance the sensitivity of NSCLC cells to chemotherapeutic agents, suggesting its potential as a therapeutic target (Chi et al., 2023).

#### 4.2.3 Ovarian cancer

In a xenograft model of ovarian cancer, administering a monoclonal antibody targeting ADAM17 effectively blocked the shedding of its substrates, which significantly reduced tumor growth (Richards et al., 2012). This finding is consistent with the role of ADAM17 in contributing to cisplatin resistance. Specifically, ADAM17 increases its activity when ovarian cancer cells are treated with cisplatin, leading to amphiregulin (AREG) shedding and subsequent EGFR signaling. Conversely, blocking ADAM17 through inhibitors, antibodies, or silencing techniques reduces AREG release and enhances the sensitivity of cancer cells to cisplatin’s apoptotic effects (Hedemann et al., 2018).

#### 4.2.4 Breast cancer

ADAM17 is involved in the process of breast cancer development and progression, shedding ligands such as TGF-α, AREG, EGFR ligands, and betacellulin and then activating EGFR which promotes breast cancer cell growth and invasion through the PI3K/AKT and Ras/MAPK signaling pathways (Shen et al., 2016). In addition, ADAM17 promotes chemotherapy resistance and tumor cell proliferation and survival by shedding EGFR ligands (e.g., Amphiregulin, AREG) (Hugendieck et al., 2023).

Collectively, these findings underscore the critical role of ADAM17 in multiple malignancies, supporting its potential as both a biomarker and a therapeutic target via genetic, antibody-based, or pharmacological inhibition.

### 4.3 Cardiovascular diseases

ADAM17 has gained increasing attention for its involvement in various cardiovascular conditions, including atherosclerosis (AS), acute myocardial infarction (AMI), cardiomyopathy, aortic aneurysm, and hypertension (Kawai et al., 2021).

#### 4.3.1 Atherosclerosis (AS)

The expression of ADAM17 in endothelial cells and myeloid cells has different implications for the development of AS (a chronic inflammatory disorder of the blood vessels). Inhibition of it in myeloid cells can lead to a significant exacerbation of AS lesions, but with a reduction in macrophage content, an increase in collagen and smooth muscle cell components, and a thickening of the fibrous cap, making the plaque more stable. In contrast, inhibition of endothelial ADAM17 expression can suppress the development of AS (Wang et al., 2020). These findings suggest that the enzyme plays a complex role in AS, and its cell-specific effects must be considered when evaluating it as a therapeutic target (van der Vorst et al., 2017).

#### 4.3.2 Acute myocardial infarction (AMI)

In patients with AMI, increased expression of ADAM17 in circulating leukocytes, along with elevated plasma TNF-α levels, is positively correlated with the severity of the disease (Satoh et al., 2008). Experimental models further demonstrate its significant upregulation during AMI, accompanied by a reduction in tissue inhibitor of metalloproteinase-3 (TIMP-3), a natural and potent inhibitor (Zheng et al., 2016). These results suggest that the upregulated ADAM17 expression significantly contributes to cardiac remodeling after AMI and highlight its potential as a therapeutic target for AMI prevention and treatment.

#### 4.3.3 Cardiomyopathy

ADAM17 mediates doxorubicin-induced cardiotoxicity by promoting TNF-α shedding, TRAF3 upregulation, and MAPK pathway activation in cardiomyocytes, following its activation via C/EBPβ induced by the chemotherapeutic agent. Importantly, ADAM17 knockdown attenuates doxorubicin-induced cardiomyopathy without compromising the antitumor efficacy of doxorubicin (Xie et al., 2024). In diabetic cardiomyopathy (DCM), the activity of ADAM17 is increased while the expression of angiotensin-converting enzyme 2 (ACE2), which has beneficial effects in ameliorating left ventricular remodeling and dysfunction, is downregulated. Genetic knockout of ADAM17 reverses these changes and attenuates cardiac fibrosis and cardiomyocyte apoptosis in DCM mice (Xue et al., 2022).

#### 4.3.4 Aortic aneurysm and hypertension

In the chronic pathological processes such as aortic aneurysm formation and ventricular remodeling, the Angiotensin II (Ang II)-ADAM17-EGFR signaling pathway plays a significant role. Inhibition of ADAM17 expression and activity can suppress the transactivation of EGFR, thereby inhibiting the progression of the disease (Takayanagi et al., 2016; Kawai et al., 2017). In hypertension, ADAM17 induces the release of TNF-α and the shedding of ACE2, making it a key point in the treatment of hypertension. In terms of vascular remodeling, ADAM17 upregulates the expression of Ang II through the transactivation of EGFR, and inhibiting ADAM17 can halt the progression of pathological changes (Yu et al., 2019; Xu et al., 2017).

### 4.4 Neurodegeneration

In the context of neurodegenerative diseases, ADAM17 is mainly implicated in the pathology of Alzheimer’s disease (AD), a progressive disorder characterized by cognitive dysfunction and amyloid β (Aβ) accumulation (Qian et al., 2016). The function of ADAM17 in AD is dichotomous, exerting both protective and detrimental effects during disease progression. On one hand, the upregulation of ADAM17 activity promotes higher levels of the neuroprotective soluble APP α fragment while decreasing Aβ generation, suggesting a potentially beneficial effect (Chandrasekaran and Bonchev, 2013). Aβ, generated through proteolytic cleavage of amyloid precursor protein (APP) by β- and γ-secretases, is widely recognized as a major contributor to neuronal impairment and, ultimately, dementia. In contrast, α-secretase selectively cleaves within APP, but its cleavage site is located outside the Aβ sequence, thereby preventing Aβ formation. ADAM17 may reduce neurotoxic APP production through its α-secretase activity, which may have an impact on the disease process (Lichtenthaler et al., 2022). On the other hand, ADAM17 contributes to neuroinflammation and neuronal injury. For example, in microglial cells, ADAM17 may facilitate neurodegeneration by promoting the release of pro-inflammatory cytokines such as TNF-α and sustaining chronic inflammatory responses. Furthermore, ADAM17 contributes to painful diabetic neuropathy (PDN) by promoting the degradation of spinal membrane ACE2, and the consequent attenuation of Ang (1–7) signaling, thereby facilitating pathological pain (Nemoto et al., 2025). These studies underscore the complex and context-dependent functions of ADAM17 in the progression of neurodegenerative disorders.

Collectively, these findings highlight ADAM17 as a potential biomarker and therapeutic target for multiple disorders. However, its clinical application faces several challenges. First, the structural similarity among ADAM family members complicates the development of highly selective inhibitors. Second, due to its broad substrate spectrum, targeting ADAM17 without disrupting essential physiological signaling remains difficult. Further research is required to develop precise strategies for selective modulation of ADAM17 activity in specific pathological contexts. In this regard, the development strategies and representative examples of ADAM17 inhibitors, including small-molecule compounds, monoclonal antibodies, and endogenous protein inhibitors, are summarized in Table 1, providing a comprehensive overview for guiding future research and drug design.

**TABLE 1:** Development strategies and representative examples of ADAM17 inhibitors.

Compound Number	Chemical Name	Mechanism	Potency	References
Small-molecule inhibitors				
- Hydroxamate-based inhibitors				
1	Marimastat	Hydroxamate compounds chelate the Zn^2+^ ion in the active site of ADAM17’s metalloprotease domain (MD)	IC_50_ = 4.75 μM	Ouvry et al. (2017)
2	Apratastat, TMI-005		IC_50_ = 0.44 nM	Ouvry et al. (2017), Boiteau et al. (2018)
3	DPC-333		IC_50_ = 17-100 nM	Ouvry et al. (2017), Boiteau et al. (2018)
4	-		IC_50_ = 8 nM	Boiteau et al. (2018)
5	-		IC_50_ = 36 nM	Boiteau et al. (2018)
6	-		N.D.	Boiteau et al. (2018)
7	Aderbasib, INCB007839		N.D.	Puthiyottil et al. (2025), Qian et al. (2016)
8	GW280264X		8 nM	Moss et al. (1997), Hundhausen et al. (2003), Hedemann et al. (2021), Saad et al. (2020)
9	INCB3619		14 nM	Fridman et al. (2007)
10	KP-457		11.1 nM	Hirata et al. (2017)
11	-		N.D.	Levin et al. (2006)
12	-		N.D.	Levin et al. (2003)
13	-		IC_50_ = 4 nM	Mazzola et al. (2008)
- Sulfonamide-based inhibitors				
14	-	The –SO_2_NH– group coordinates with catalytic Zn^2+^ in the active site of MD; fits ADAM17’s unique “L-shaped” S1' hydrophobic pocket.	N.D.	Levin et al. (2003)
15	-		IC_50_ = 3 nM	Zhang et al. (2004a)
16	TMI-1		IC_50_ = 8.4 nM	Maskos et al. (1998), Levin et al. (2006)
17	-		IC_50_ = 20 nM	Levin et al. (2006)
18	-		IC_50_ = 67 nM	Levin et al. (2006)
19	TMI-2		IC_50_ = 2 nM	Zhang et al. (2004b)
20	-		N.D.	Nuti et al. (2010)
21	-		N.D.	Nuti et al. (2013)
22	JG26		IC_50_ = 1.9 nM	Park et al. (2006)
23	-		IC_50_ = 1.5 nM	Park et al. (2006)
24	-		IC_50_ = 2.2 nM	Condon et al. (2007)
- γ-Lactam inhibitors				
25	IK682	Carbonyl group coordinates with catalytic Zn^2+^; the γ-lactam scaffold fit the S1' pocket.	N.D.	Duan et al. (2002), Niu et al. (2006)
26	-		IC_50_ = 0.42 nM	Duan et al. (2003)
- Carboxylate inhibitors				
27	-	–COO^-^ group chelates Zn^2+^; Fits into S1' pocket.	Ki = 143 nM	Guo et al. (2009)
28	TAPI-1		IC_50_ = 8.09 nM	Maskos et al. (1998), Slack et al. (2001), Aljohmani et al. (2022), Lingott et al. (2009)
29	TAPI-2		IC_50_ = 18 nM	Maskos et al. (1998), Lingott et al. (2009)
- Hydantoin-based inhibitors				
30	-	Carbonyl and/or NH groups interact with Zn^2+^; Hydantoin ring can partially occupy S1' pocket.	Ki = 6 μM	Yu et al. (2010a)
31	-		N.D.	Yu et al. (2010a)
32	-		Ki = 23 nM	Yu et al. (2010a)
33	-		Ki = 4 nM	Yu et al. (2010a)
34	-		Ki = 5 nM	Yu et al. (2010a)
35	-		Ki = 0.4 nM	Yu et al. (2010a)
36	-		Ki = 0.8 nM	Yu et al. (2010a)
37	SCH900567		N.D.	Ren et al. (2014)
38	-		Ki = 0.7 nM	Tong et al. (2017a)
39	-		Ki = 0.5 nM	Tong et al. (2017a), Tong et al. (2017b)
40	-		Ki = 0.4 nM	Tong et al. (2017a)
41	-		Ki = 0.62 nM	Tong et al. (2017b)
42	-		Ki = 0.3 nM	Tong et al. (2017b)
43	-		Ki = 6 nM	Yu et al. (2010b)
44	-		Ki = 0.8 nM	Yu et al. (2010b)
45	-		N.D.	Yu et al. (2010b)
- Tartrate-based inhibitors				
46	-	The tartrate structure chelates the Zn^2+^ to form a bidentate or multidentate ligand; the hydrophobic group binds to the S1′ pocket.	Ki = 400 nM	Rosner et al. (2010)
47	-		Ki = 400 nM	Rosner et al. (2010)
48	-		Ki = 96 nM	Rosner et al. (2010)
49	-		IC_50_ = 8 nM	Rosner et al. (2010)
50	-		Ki = 0.8 nM, cell IC_50_ = 886 nM, AUC = 2949 nM.h	Li et al. (2010)
51	-		Ki = 5 nM, cell IC_50_ = 3016 nM, AUC = 134,267 nM.h	Li et al. (2010)
52	-		Ki = 1nM, cell IC_50_ = 416 nM, AUC = 1133 nM.h	Li et al. (2010)
53	-		N.D.	Li et al. (2010)
54	-		Ki = 45 nM	Li et al. (2010)
55	-		Ki = 20 nM	Li et al. (2010)
56	-		Ki = 2 nM	Li et al. (2010)
57	-		Ki = 4 nM	Li et al. (2010)
58	-		Ki = 0.85 nM	Li et al. (2010), Banchelli et al. (2013)
59	MBET306		N.D.	Li et al. (2010), Banchelli et al. (2013)
- Other non-hydroxamate-based small-molecule inhibitors				
60	9c	Coordinate the catalytic Zn^2+^ via thiols, carboxylates, or heterocyclic groups; exploit hydrophobic or extended P1' groups to engage ADAM17’s S1' pocket (sometimes extending to S3′)	N.D.	Pu et al. (2015)
61	-		N.D.	Duan et al. (2007)
62	-		N.D.	Duan et al. (2007)
63	-		IC_50_ = 2 nM	Duan et al. (2007)
64	-		Ki = 28 nM	Govinda Rao et al. (2007)
65	-		Ki = 10 nM	Govinda Rao et al. (2007)
66	-		Ki = 10 nM	Govinda Rao et al. (2007)
67	-		IC_50_ = 8.2 nM	Park et al. (2009)
68	-		IC_50_ = 2.5 nM	Park et al. (2009)
69	-		IC_50_ = 0.14 μM	Park et al. (2009)
70	-		IC_50_ = 0.14 μM	Park et al. (2009)
71	-		IC_50_ = 0.37 μM	Park et al. (2009)
72	-		IC_50_ = 0.08 μM	Park et al. (2009)
73	ZLDI-8		IC_50_ = 6.85 μM	Li et al. (2018), Lu et al. (2019), Lu et al. (2020), Xu et al. (2024)
74	NY-2		N.D.	Zhang et al. (2023)
75	SN-1		N.D.	Fujita et al. (1996), Tateishi et al. (2021), Radwan et al. (2019)
76	SN-4(Nps)2		N.D.	Tateishi et al. (2021)
77	SN-4		IC_50_ = 3.22 μM	Toma et al. (2024)
78	JTP-96193		IC_50_ = 5.4 nM	Maekawa et al. (2019)
Monoclonal antibodies				
-	A300, A309, A318	Binds to the disintegrin domain, with enhanced affinity for the disintegrin–EGF-like conformation	-	Trad et al. (2011)
-	A300E	Binds to the membrane-proximal cysteine-rich domain; bispecific anti-CD3 promotes anticancer T cell activity	IC_50_ = 0.7 mg/mL	Yamamoto et al. (2012), Trad et al. (2013)
-	A9(B8)	A9 binds the MD; B8 binds an allosteric site outside the catalytic cleft; Reduces the shedding of ErbB ligands and suppresses the progression of pancreatic cancer	Ki = 0.33 nM; K_d_ = 0.22 nM; IC_50_ = 0.22 nM (human); 0.25 nM (mouse)	Yang et al. (2019), Ye et al. (2017)
-	D1 (A12)	Binds the metalloprotease domain and blocks the release of various ADAM17 substrates	4.7 nM	Zhang et al. (2004a), Zhang et al. (2004b), Zhang et al. (20012)
-	MEDI3622	Binds the MPD and inhibits cleavage of EGFR ligands	K_d_ = 39 pmol/L (human); 132 pmol/L (mouse)	Rios-Doria et al. (2015)
-	D8P1C1	Binds to the MD and inhibits its proteolytic activity	IC_50_ = 0.4 nM	Saha et al. (2022)
-	C12	Binds to the MPD and hinders the cleavage of EGFR ligands	∼25 nM (BLI)	Saha et al. (2024)
Endogenous protein inhibitors				
-	TIMP3	Binds ADAM17 through defined hydrophobic and loop-mediated interactions rather than classical Zn^2+^ chelation	Ki = 3.61 nM	Wisniewska et al. (2008), Carreca et al. (2020)

^*^N.D., refers to not detected.

## 5 Small molecule inhibitors of ADAM17

The catalytic domain of ADAM17 includes a zinc-binding motif (HEXXHXXGXXH) where zinc ions are coordinated by three histidine residues. An effective ADAM17 inhibitor should be able to dislocate the substrates from this site and bind to the zinc ions, thereby interfering with the enzymatic activity. A crystallographic structural analysis of a hydroxamate inhibitor bound to ADAM17 reveals that the enzyme’s active site exhibits several similarities with matrix metalloproteinases (MMPs) (Maskos et al., 1998). These shared features include a catalytic zinc ion coordinated by three histidine residues, a hydrophobic S1′ pocket near the metal center, and hydrogen bond donors and acceptors, including the carboxylate moiety of E406 and the backbone amide of G349. However, one unique characteristic of ADAM17 is the presence of a tunnel linking the S1′ and S3′ pockets, forming an expanded cavity. This structural difference suggests that selective inhibition could be achieved by introducing bulky substituents at the P1′ position, which would be incompatible with the more confined S1′ pocket found in MMPs. Developing ADAM17-specific inhibitors is particularly important, as they have the potential to circumvent the musculoskeletal adverse effects associated with broad-spectrum and partially selective MMP inhibitors (Rosenbaum et al., 2005; Wojtowicz-Praga et al., 1998). Hydroxamic acids are a common zinc-binding motif that has been widely used as a zinc-binding ligand by researchers due to its potency and strong affinity for zinc ions. However, these acids often exhibit poor absorption due to high renal clearance. Additionally, they may undergo metabolic liabilities *in vivo*, such as O-glucuronidation and hydrolysis, which can produce toxic by-products like carboxylic acids and hydroxylamine (Dai et al., 2011). Also, its strong zinc binding leads to a loss of selectivity. To mitigate the toxicity and side effects linked to the hydroxamate group while enhancing bioavailability, efforts to develop new ADAM17 inhibitors have shifted toward non-hydroxamate small molecules (Calligaris et al., 2021). Several non-hydroxamate ADAM17 inhibitors have been identified, in which alternative zinc-binding groups (ZBGs) replace the conventional hydroxamate moiety, such as a pyrimidinetrione (Duan et al., 2005), hydantoin (Sheppeck et al., 2007a), triazolone, imidazolone (Sheppeck et al., 2007b) and triazolethione (Gilmore et al., 2007). In recent years, monoclonal antibodies have emerged as a novel type of ADAM17 inhibitors, which exhibit high selectivity and therapeutic potential by specifically binding to ADAM17 and blocking its function, demonstrating the advantages of high specificity, long-lasting efficacy, and low toxicity.

### 5.1 Hydroxamate-based inhibitors

**FIGURE 3:**
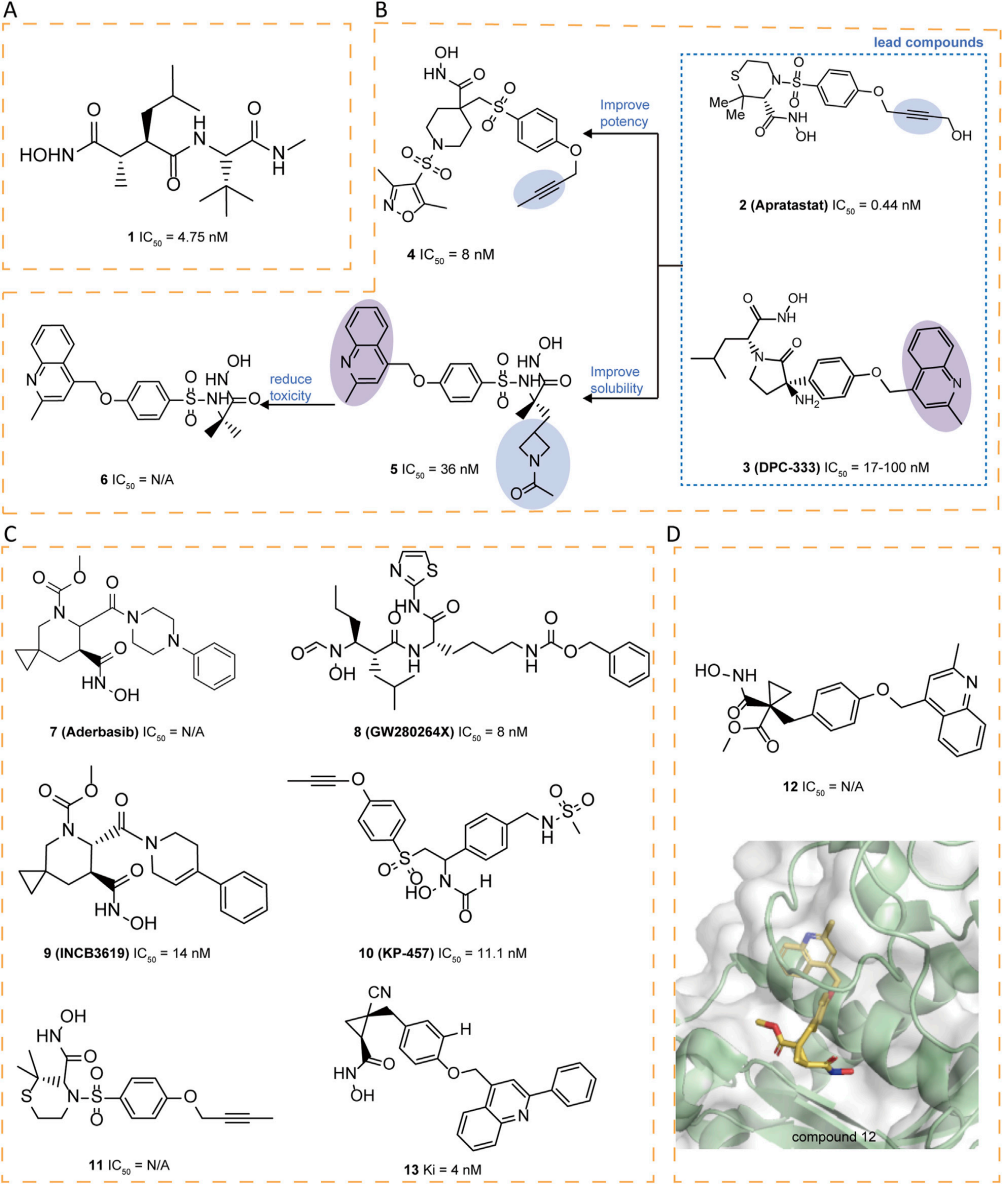
**(A)** Chemical structures of compounds 1. **(B)** Chemical structures and structure–activity relationships of compounds 2-6. **(C)** Chemical structures of compounds 7-11,13. **(D)** Chemical structures and crystal structure of catalytic domain of ADAM17 combined with compound 12 (PDB ID: 3EDZ).

**1** (Marimastat) and **2** (Apratastat/TMI-005), the first hydroxamic acid-based broad-spectrum matrix metalloproteinase inhibitors with limited selectivity, contain an alkyne group that enables copper-catalyzed azide-alkyne cycloaddition (CuAAc) with azide-containing molecules (Figures 3A,B). Although **2** exhibited no side effects in Phase I clinical trials, it was discontinued in Phase II due to a lack of sufficient efficacy (Shu et al., 2011).

A literature highlighted the importance of an alkyne group as in **2** and a methyl quinoline group as in **3** (DPC-333) in the S1′ pocket of ADAM17, as these groups significantly enhance the potency and selectivity of the inhibitors (Ouvry et al., 2017). Taking **2** and **3** as the leads, novel cyclic linkers were explored to tether hydroxamate as the zinc-binding group, with **4** and **5** emerging as the most promising candidates. Compound **4** is more potent but showed suboptimal solubility. Compound **5** displayed improved solubility but carried a genotoxicity warning. To further enhance potency and selectivity while reducing genotoxicity, sulfonamide-hydroxamate derivatives were explored by substituting the N-acetyl group with an azetidine nitrogen and methylating the sulfonamide nitrogen. The optimal balance was achieved with azetidine derivative **6**, derived from **5**, which exhibited increased potency, reduced toxicity and good solubility (Figure 3B) (Boiteau et al., 2018).

Compound **7** (Aderbasib, INCB007839), originally developed by Incyte Corporation, is a dual-target small molecule inhibitor of ADAM10 and ADAM17. Its core sulfonamide-hydroxamate scaffold endows it with potent antineoplastic activity. **7** has been investigated for its therapeutic potential in various cancers, including HER2^+^ breast cancer, diffuse large B-cell non-Hodgkin lymphoma, gliomas, and other malignancies (Witters et al., 2008). The sulfonamide moiety binds to its S1′ pocket, enhancing inhibitor selectivity, while the hydroxamate group effectively chelates the zinc ion at the active site, leading to potent enzyme inhibition. Additionally, the introduction of an azetidine ring in the molecule not only improves solubility but also reduces off-target effects and toxicity. However, side effects such as thromboembolism, pain, and infection have been reported. A study shows that when **7** is used in combination with PEPDG278D (a recombinant human protein), it can significantly enhance tumor growth inhibition, particularly in tumors that overexpress EGFR ligands (Yang et al., 2022). **8** (GW280264X) potently blocks ADAM17 with IC_50_ of 8.0 nM (Hundhausen et al., 2003). The core structure of **8** mainly consists of a hydroxamate group and a biaryl system that significantly enhances its binding affinity and selectivity through interaction with the S1′ pocket of ADAM17 (Moss et al., 1997). **8** enhances the antitumor effect of cisplatin on ovarian cancer cells through inhibition of ADAM17 and exhibits growth inhibitory effects in a lung adenocarcinoma cell model (Hedemann et al., 2021; Saad et al., 2020) **9** (INCB3619) is a selectively potent and orally bioavailable inhibitor (IC_50_ = 14 nM), which demonstrated synergy with clinically relevant cancer therapeutics without exhibiting significant or compounding toxicities, including fibroplasia (Fridman et al., 2007). **10** (KP-457), featured with a reverse-hydroxamate structure, demonstrated high selectivity for ADAM17(IC_50_ = 11.1 nM) in cell-free enzyme assays. compound **10** can enhances the yield of functional platelets originating from human induced pluripotent stem cells (hiPSCs) through the inhibition of ectodomain shedding of platelet glycoprotein Ibα (GPIbα). Regarding safety, the compound showed no evidence of genotoxic or systemic toxic effects when given intravenously to dogs at doses of up to 3 mg/kg daily over a period of 4 weeks (Hirata et al., 2017). This suggests that **10** has a favorable safety profile, making it promising for further development in the treatment of diseases related to ADAM17 dysregulation (Figure 3C).

Compounds like thiomorpholine hydroxamate **11** (Levin et al., 2006) and cyclopropyl hydroxamate **12** (Levin et al., 2003) have emerged as strong ADAM17 inhibitors, demonstrating favorable selectivity against many MMPs. However, a certain degree of activity against certain MMPs, notably MMP-3 and MMP-7, has been noted. The crystal structure reveals the binding mode of **12** with ADAM17, identifying the S3′ pocket as a potential area for optimization (Figure 3D). Based on this insight, researchers designed various cyclopropyl hydroxamic acid derivatives and explored structure-activity relationships by modifying the substituents in the S3′ pocket. Different substituents significantly influenced the compounds’ affinity for ADAM17 were observed. Compound **13** exhibited a reasonable Area Under the Curve (AUC) and distinguished itself as having the best Pharmacokinetic (PK) profile (Mazzola et al., 2008) (Figure 3C).

### 5.2 Sulfonamide-based inhibitors

**FIGURE 4:**
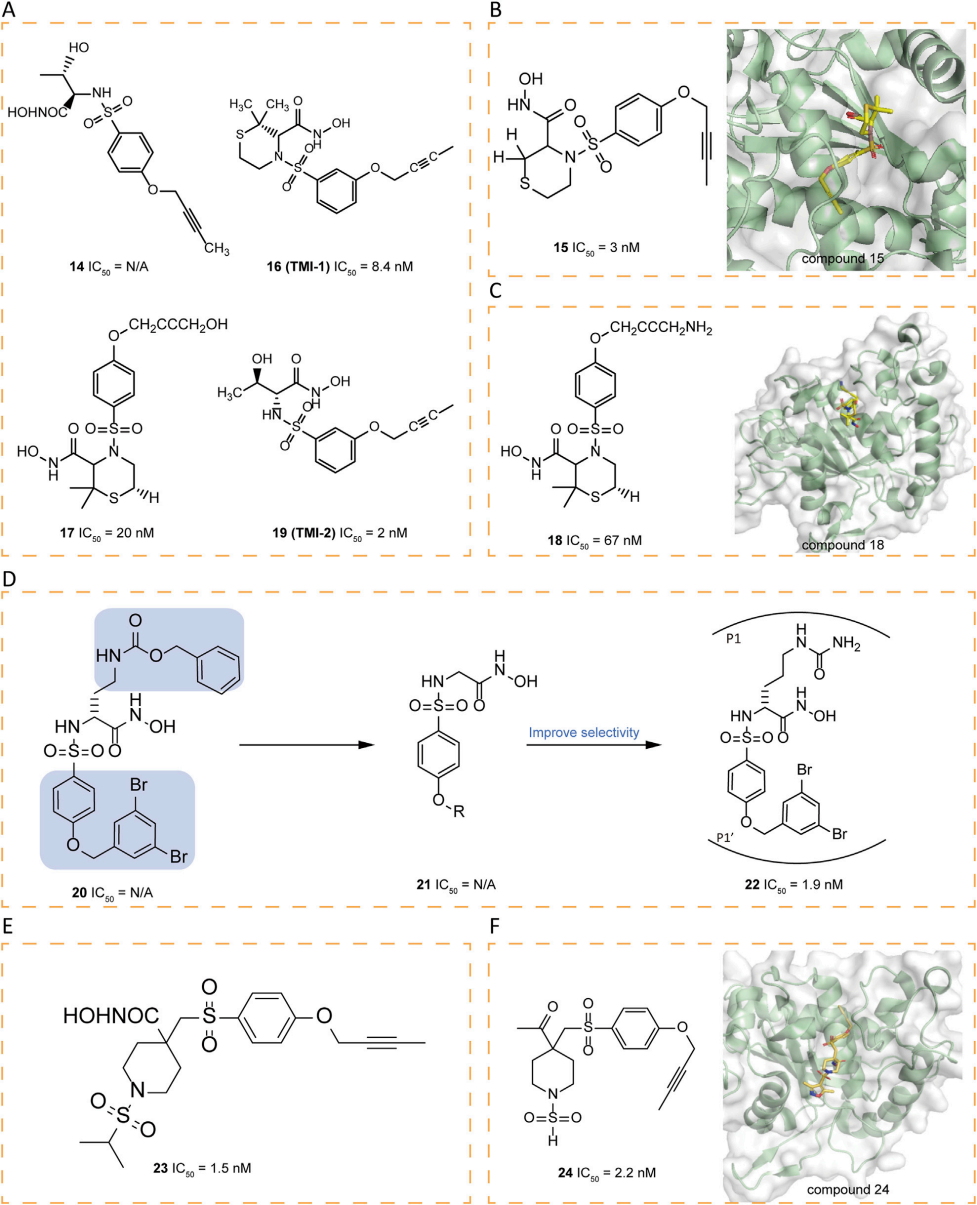
**(A)** Chemical structures of compounds 14,16,17,19. **(B)** Chemical structures and crystal structure of catalytic domain of ADAM17 combined with compound 15 (PDB ID: 1ZXC). **(C)** Chemical structures and crystal structure of catalytic domain of ADAM17 combined with compound 18 (PDB ID: 2A8H). **(D)** Chemical structures and structure–activity relationships of compounds 20-22. **(E)** Chemical structures of compounds 23. **(F)** Chemical structures and crystal structure of catalytic domain of ADAM17 combined with compound 24 (PDB ID: 2I47).

Researchers synthesized a series of sulfonamide hydroxamate derivatives based on acyclic α-amino acids and tested their inhibitory effects. The study found that most of these compounds have a strong inhibitory effect, and some compounds show higher selectivity for ADAM17 inhibition over MMP-1 and MMP-9. In particular, compound **14** demonstrated good oral activity against LPS-stimulated TNF-α in mouse models and also showed efficacy in preventive collagen-induced arthritis models (Figure 4A) (Levin et al., 2003). The structure-activity relationship (SAR) of a series of potent sulfonamide hydroxamate ADAM17 inhibitors with a butynyloxy P1' group was continuously investigated, leading to the acquisition of the X-ray crystal structure of compound **15** in complex with ADAM17 (Figure 4B). A dual ADAM17/MMP inhibitor, compound **16** (TMI-1), demonstrated superior *in vitro* potency against ADAM17 and in cellular assays, as well as oral bioactivity in an *in vivo* model of TNF-α production and a collagen-induced arthritis model (Zhang et al., 2004a). The design of the P′ moiety in **16** took into account the distinct dimensions and contours of the S1′ and S3′ pockets (Maskos et al., 1998). Unfortunately, **16** was hindered from progressing to clinical candidacy due to its low solubility and bioavailability. Attempts to optimize the bioavailability and potency of the thiomorpholine sulfonamides involved modifications to the P1' terminus and appending substituents on the thiomorpholine ring, ultimately leading to the development of clinical candidates. Compound **17** exhibits activity comparable to that of **16** in of rheumatoid arthritis (RA) models, while demonstrating enhanced solubility and PK properties. Interestingly, the amine moiety of **18** is within hydrogen bonding distance to both a glutamate and valine residue in the S3′ pocket, but is less active against ADAM17 than **16** (Figure 4C) (Levin et al., 2006).

The butynyloxy P1′ group is positioned within the channel that links the S1′ and S3′ enzyme subsites, thereby boosting the inhibitory effectiveness against both the enzyme and at the cellular level. Subsequent refinements to 16 have led to the discovery of **19** (TMI-2), a derivative that incorporates an acyclic sulfonamide framework equipped with a butynyloxy P1′ moiety. This analog stands out as a highly potent, reversible, and selective inhibitor (IC_50_ = 2 nM). Compared to **16**, an enhanced selectivity profile is obtained over various MMPs. The key distinction between these two compounds lies in their P1 group. The switch from the cyclic thiomorpholine P1′ group in 16 to a linear NH-sulfonamide structure has been instrumental in enhancing selectivity (Zhang et al., 2004b).

Arylsulfonamide hydroxamates, exemplified by compound **20**, have demonstrated nanomolar-level potency without exhibiting toxicity (Nuti et al., 2010). The fine-tuning of the benzyloxyaryl segment in compound **21** has given rise to derivatives that bear the 3,5-dibromobenzyloxyphenyl moiety, which confers heightened selectivity. The subsequent introduction of an amide chain at the α-position of the hydroxamate (P1 group) has resulted in **22** (JG26), which boasts significantly amplified selectivity (Figure 4D) (Nuti et al., 2013). Drawing on the previously reported 4,4′-piperidine β-sulfone hydroxamate inhibitor **23** (IC_50_ = 1.5 nM), research endeavors have concentrated on exploring alkyl sulfonamide variants, N-arylation of heteroaryl piperidines, and heterocyclic sulfonamides to refine selectivity (Figure 4E) (Park et al., 2006). Researchers have pinpointed the β, β′-disubstitution of the substituent on the P1 sulfonamide of the piperidine ring as a structural feature that enhances selectivity. Consequently, they synthesized a series of sulfamide derivatives. Among them, compound **24**, adorned with a 3,5-dimethyl isoxazole group, has shown a remarkable selectivity for ADAM17 versus MMP-2 and MMP-13, with a selectivity ratio exceeding 300-fold (Figure 4F) (Condon et al., 2007).

### 5.3 γ-Lactam inhibitors

**FIGURE 5:**
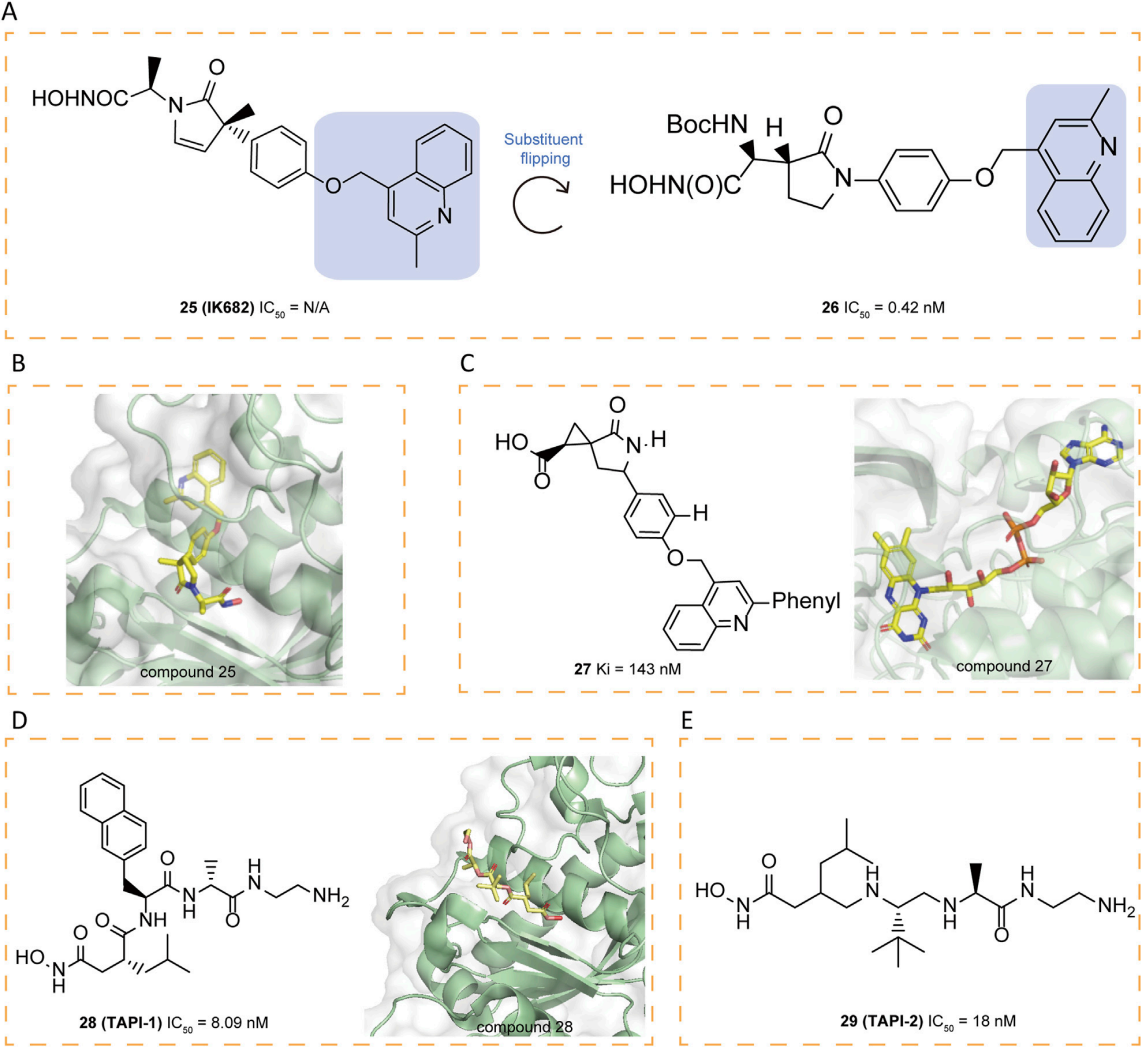
**(A)** Chemical structures and structure–activity relationships of compounds 25-26. **(B)** Crystal structure of catalytic domain of ADAM17 combined with compound 25 (PDB ID: 2FV5). **(C)** Chemical structures and crystal structure of catalytic domain of ADAM17 combined with compound 27 (PDB ID: 3EWJ). **(D)** Chemical structures and crystal structure of catalytic domain of ADAM17 combined with compound 28 (PDB ID: 2DDF). **(E)** Chemical structures of compounds 29.

In order to further improve selectivity of inhibitors, a series of γ-lactam hydroxamates were designed (Duan et al., 2002). Building on the structural framework of MMP inhibitors, a rational exploration of P1′-S1′ interactions led to the 3,5-disubstituted benzyl ether as a key P1′ group conferring selectivity for ADAM17. In order to enhance the free fraction in serum and cell membrane permeability, a polar bioisostere, **25**(IK682), 2-methylquinolinylmethoxy, was introduced to improve the pharmacokinetic properties of the compound, particularly its activity in the whole blood assay. It exhibited excellent selectivity relative to MMPs. The X-ray crystal structure analysis reveals the tight binding of **25** to ADAM17, with **25** forming multiple binding points at the S1 and S3 sites of ADAM17 (Figure 5A). Notably, the quinolinyl group of **25** is partially located in the S3′ pocket, a structural feature that necessitates a conformational change in the ADAM17 protein: the loop spanning from amino acid Alanine 349 to Glycine 442 shifts away from the enzyme’s central structure by up to 5 Å to facilitate the integration of the quinolinyl moiety. This conformational change, particularly the movement of the specificity loop, significantly enhances the high-affinity binding of **25** to ADAM17 and contributes to the formation of a more stable complex (Figure 5B).

Liquid chromatography/mass spectrometry (LC/MS) analysis, after separating the ADAM17/**25** complex under denaturing conditions, confirms that the tight binding between **25** and ADAM17 is not due to covalent interactions. The hydroxamate moiety of **25** engages with the zinc ion at ADAM17’s active site and forms coordination bonds with both the carboxylate group of Glutamate 406 and the carbonyl oxygen of Glycine 349. Furthermore, the carbonyl group of the pyrrolidine ring in compound **25** interacts with the amide nitrogens of Leucine 348 and Glycine 349 through hydrogen bonding, and the methyl group attached to the pyrrolidine engages in van der Waals interactions with the carbonyl oxygen of Glycine 346. These non-covalent interactions collectively contribute to the specificity and stability of the interaction between **25** and ADAM17 (Niu et al., 2006). In an effort to expand the range of ADAM17 inhibitors, researchers explored the possibility of flipping the γ-lactam ring, which led to the identification of the N-hydroxy-2-(2-oxo-3-pyrrolidinyl) acetamide scaffold as a new framework. By incorporating a selective (2-methyl-4-quinolinyl)methoxy P1′ group, researchers developed a series of highly effective inhibitors, among which compound **26** exhibited the greatest potency, achieving an IC_50_ of 0.42 μM in the whole-cell assay (WBA) (Duan et al., 2003).

### 5.4 Carboxylate inhibitors

In general, carboxylate functions as a weaker zinc-binding ligand compared to hydroxamate. Within the cyclopropyl series, replacing hydroxamate with carboxylate leads to a drastic loss of activity and potency reduction. In the spirocyclopropyl series, however, the decrease in the inhibition constant (Ki) is more moderate, ranging from only 5–100-fold upon substitution of carboxylate for hydroxamate. The crystallographic structure of **27** bound to ADAM17 illustrates its carboxylate group coordinates with the zinc ion in a bidentate manner, maintaining O–Zn distances of 1.9 Å and 2.5 Å, respectively (Figure 5C). Furthermore, the weaker zinc-binding oxygen of the carboxylate engages in a hydrogen bond with the side chain of Glu406, and the carboxylic acid is anticipated to be protonated. Additionally, the lactam’s carbonyl oxygen forms a hydrogen bond with the backbone NH of Leu348. A crystalline water molecule serves as a hydrogen-bonding bridge between the lactam NH and the carbonyl oxygen of Gly346 (Guo et al., 2009).

Compound **28** (TAPI-1) exhibits the ability to inhibit MMPs and prevent cytokine receptor shedding with an IC_50_ of 8.09 μM (Slack et al., 2001). Recent research has demonstrated that **28** effectively suppresses ADAM17 activation during *Pseudomonas aeruginosa* infection (Aljohmani et al., 2022). To further investigate its binding characteristics, Bienstein et al. conducted molecular docking simulations comparing the positioning of **28** and **29** (TAPI-2) within ADAM17and revealed that **28** occupies into the substrate-binding site adjacent to the bound zinc ion (Maskos et al., 1998; Lingott et al., 2009). The hydroxamate group of **28** coordinates with the zinc ion, while its isobutyl moiety sits in the deep hydrophobic S1′ pocket and the long chain on the opposite side of the hydroxamate group extends into the S3′ binding pocket. The binding site and orientation of **28** within the catalytic domain of ADAM17 closely resemble those of **29**, co-crystallized with ADAM17 (Figures 5D,E) (Lingott et al., 2009). Structurally, ADAM17 is characterized by a shallower hydrophobic S1′ pocket and a deep hydrophobic S3′ pocket, interconnected by water channels. These features facilitate binding, allowing isobutyl group of **28** to occupy the S1′ pocket, while the long-chain substituent extends into the S3′ site (Maskos et al., 1998). Furthermore, the autoproteolysis of ADAM17 at the Y352-V353 cleavage site, situated within a flexible loop adjacent to the P-side of the active site, exhibits a concentration-dependent behavior, indicative of a bimolecular reaction mechanism. Specificity studies using surrogate peptides have identified mutations (V353G and V353S) that significantly enhance resistance to autoproteolysis (Ingram et al., 2006). Eltrombopag, a thrombopoietin receptor agonist, was recently identified as a non-targeted inhibitor of ADAM17, with its carboxyl group exhibiting strong affinity for the catalytic Zn^2+^ ion, highlighting the functional relevance of carboxylate groups in mediating ADAM17 inhibition (Puthiyottil et al., 2025).

### 5.5 Hydantoin-based inhibitors

**FIGURE 6:**
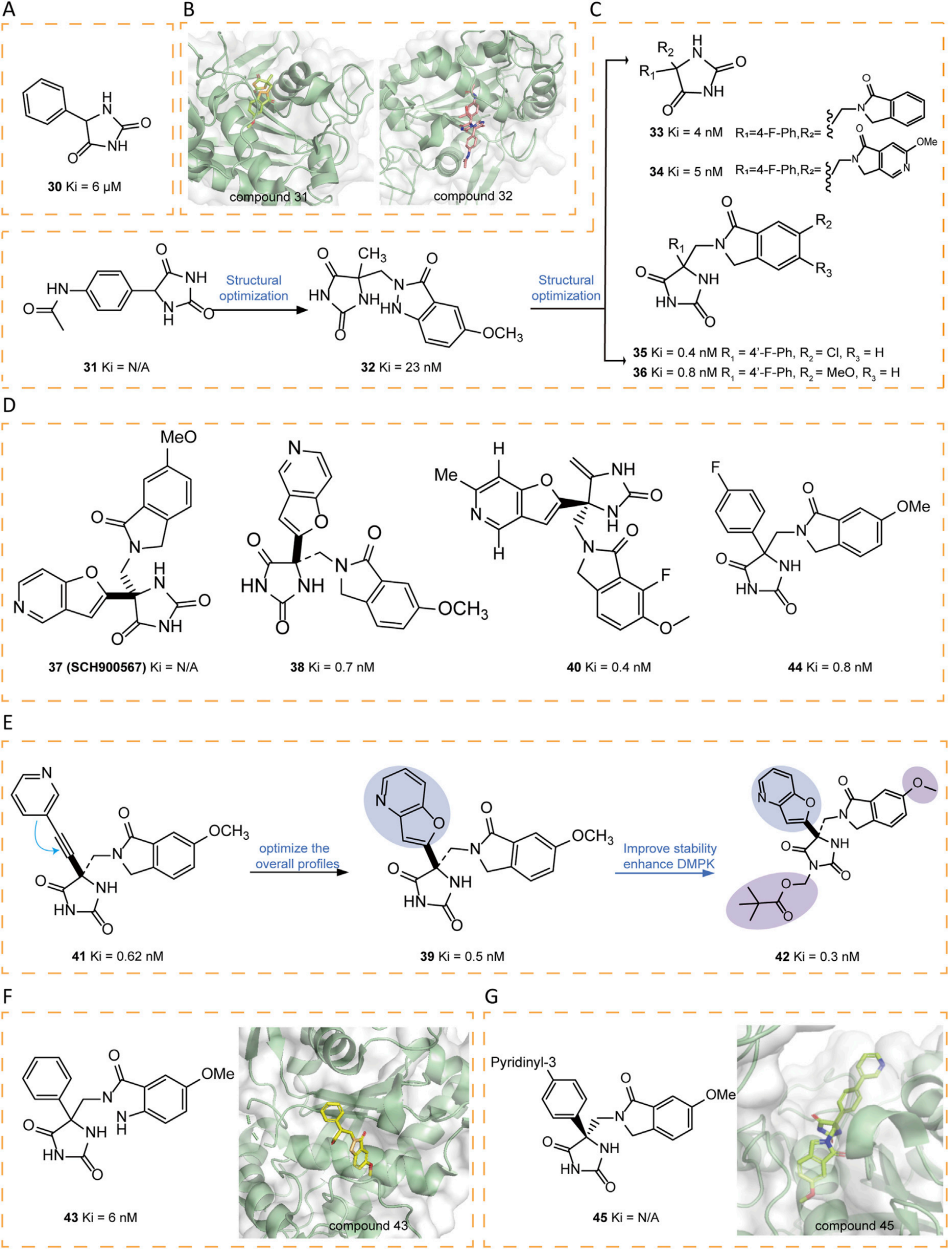
**(A)** Chemical structures of compound 30. **(B)** Crystal structure of catalytic domain of ADAM17 combined with compound 31 (PDB ID: 3L0T) and compound 32 (PDB ID: 3L0V). **(C)** Chemical structures and structure–activity relationships of compounds 31-36. **(D)** Chemical structures of compounds 37-38,40,44. **(E)** Chemical structures and structure–activity relationships of compounds 39,41-42. **(F)** Chemical structures and crystal structure of catalytic domain of ADAM17 combined with compound 43 (PDB ID: 3LE9). **(G)** Chemical structures and crystal structure of catalytic domain of ADAM17 combined with compound 45 (PDB ID: 3LEA).

A series of innovative hydantoin derivatives was designed and synthesized as structural substitutes for hydroxamate-based ADAM17 inhibitors (Sheppeck et al., 2007a). The features contributed to the activity of these compounds are: the strict requirement for unsubstituted hydantoin nitrogen atoms, the presence of the unnatural (5R) stereochemistry, and the incorporation of a hydrogen bond acceptor attached to a properly functionalized hydrophobic P′ side chain, which effectively interacts with the S1 pocket (Sheppeck et al., 2007a). Compound **30** was identified as weak inhibitors (Figure 6A). The crystal structure of the hydantoin-containing compound **31** shows that the active site accommodates two molecules, one positioned in the S1′ pocket and the other in the S1 region. In contrast to hydroxamates and tartrates, which coordinate zinc in a bidentate or tridentate form, **31** exhibited monodentate zinc binding through its S1 hydantoin nitrogen. The amide nitrogen engages with the carbonyl oxygen of Gly349 and the adjacent carbonyl oxygen, forming a bidentate hydrogen bond to the side chain of Glu406. The phenyl group resides within the hydrophobic non-prime site, which is defined by the side chains of Thr347, Leu350, and Lys315, Val314. Meanwhile, the former molecule forms hydrogen bonds between the hydantoin carbonyl and the backbone NH groups of Leu348 and Gly349, with the phenylacetamide moiety embedded within the S1′ pocket. Based on the binding mode, a novel scaffold was designed to preserve the zinc-binding capability of hydantoin while simultaneously engaging in key interactions, including hydrogen bonding with the backbone NH groups of Leu348 and Gly349, as well as effectively occupying the S1′ pocket. Among the designed compounds, **32** exhibited the most favorable binding characteristics (Figure 6B). Compound **32** served as the foundation for synthesizing various analogs, primarily 1H-indazol-3(2H)-one derivatives, isoindolin-1-one analogs, and analogs with modified ring systems. Among these, compounds **33–36** demonstrated strong inhibitory activity (Figure 6C) (Yu et al., 2010a).

Compound **37** (SCH900567), a hydantoin derivative with ADAM17 inhibitory activity, has been explored as a potential treatment for rheumatoid arthritis. To support initial drug metabolism and pharmacokinetic studies, [(3)H]SCH900567 was synthesized. Additionally, the bioanalytical team required a stable isotope-labeled analog, [(13)C3, (15)N]SCH900567, to serve as an internal standard for the development of an LC-MS/MS analytical method. In addition, the Drug Metabolism and Pharmacokinetics group sought this labeled compound for a potential microdose study. The synthesis of [(13)C3, (15)N]SCH 900567 was achieved through a seven-step sequence from commercially available materials with a total yield of 2.6% (Figure 6D) (Ren et al., 2014).

Tong et al. identified a series of hydantoin-based ADAM17 inhibitors characterized by a fused bi-heteroaryl moiety, exhibiting sub-nanomolar Ki values, strong activity in human whole blood assays (hWBA), and enhanced drug metabolism and pharmacokinetic (DMPK) properties in comparison with previous compounds. Among these, compounds **38–40** demonstrated an optimal balance of hWBA activity and rapid rat AUC values (Tong et al., 2017a). The incorporation of polar functional groups, especially those possessing hydrogen bond donor properties and basicity, into the aza-benzofuran ring markedly improved hWBA activity. Additionally, specific substituents such as methyl and fluorine atoms contributed to improved pharmacokinetic properties. Compound **41** represents a novel series of acetylene-based hydantoin inhibitors targeting ADAM17, exhibiting enhanced Ki values and hWBA performance. In order to optimize the overall profile of **41**, the acetylene moiety was cyclized with the adjacent phenyl/pyridine ring, yielding benzofuran and aza-benzofuran derivatives. Among them, compound **42**, the pivalate prodrug of **39**, was developed to maintain a stable neutral form and exhibited superior DMPK properties compared to its parent compound (Figures 6D,E) (Tong et al., 2017b).

Another optimization strategy, focusing on the SAR of the non-prime region of active site of ADAM17, identified noval hydantoin-based inhibitors, such as **43–45**. The crystal structure of **43** bond to ADAM17 active site revealed that the non-prime region was more accommodating to substitutions than initially anticipated (Figure 6F). This insight prompted the researchers to explore the substitution possibilities at the C4 position of the hydantoin core, leading to the synthesis of a series of biaryl-substituted hydantoin compounds with sub-nanomolar Ki, good rat PK, and selectivity. The crystal structure of **45** bound to ADAM17 further validated the design strategy, illustrating how the biaryl group interacted with the protein’s active site, including hydrophobic contacts with key residues such as Val314, Lys315, Thr347, and Leu350 (Figure 6G) (Yu et al., 2010b).

### 5.6 Tartrate-based inhibitors

**FIGURE 7:**
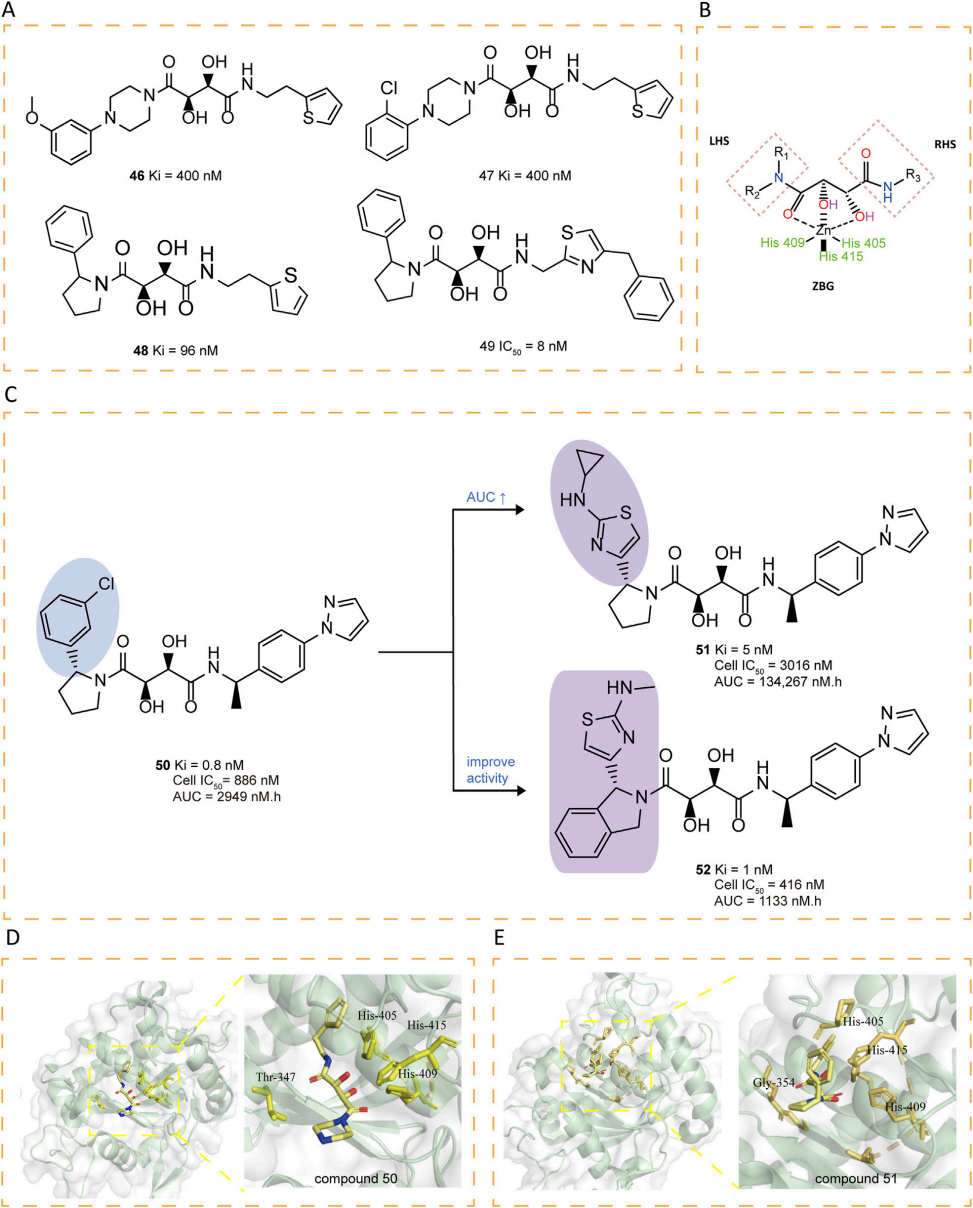
**(A)** Chemical structures of compounds 46-49. **(B)** Tridentate chelation of the zinc atom with the tartrate core. **(C)** Chemical structures and structure–activity relationships of compounds 50-52. **(D)** Crystal structure of catalytic domain of ADAM17 combined with compound 50 (PDB ID: 3KMC). **(E)** Crystal structure of catalytic domain of ADAM17 combined with compound 51 (PDB ID: 3KME).

Rosner et al. utilized the Automated Ligand Identification System (ALIS) to screen a proprietary mixture-based combinatorial library, leading to the first identification of bis-amides of L-tartaric acid inhibitors (compounds **46–47**), which feature a tartrate core connecting right-hand side (RHS) and left-hand side (LHS) substituents through amide linkages (Figures 7A,B). To further optimized these compounds, the researchers employed various strategies, including SAR exploration, 2-aryl pyrrolidine optimization, secondary P1′ amide optimization, tartrate core modifications. The crystal structure of compound **48** revealed that it retained zinc coordination and prime site interactions similar to those observed in compound **47** while introducing a new face-edge interactivity between the 2-phenylpyrrolidine and His415. Further modifications, such as replacing the thiophene ring with a thiazole, yielded active derivatives (e.g., compound **49)**, and a methylene-linked system preference (Rosner et al., 2010).

The presence of a tertiary amide on the LHS and a secondary amide on the RHS of the tartrate core seems essential for ADAM17 inhibitory activity. Comprehensive SAR studies revealed that among various 2-phenylpyrrolidine LHS fragments, (R)-1-(4-(1H-pyrazol-1-yl)phenyl)ethanamine identified as the optimal RHS amine (e.g., compound **50**), showing strong binding affinity, moderate cellular activity, as well as acceptable oral PK in rats (Li et al., 2010). Subsequently, attention was turned to refining the LHS amine building block, with the aim of improving its oral PK profile and biochemical efficacy (Dai et al., 2011). The compounds with mono-alkyl N-substitutions generally retained high binding affinities, with cyclopropyl-substituted compound **51** exhibiting notably high AUCs and sustained drug concentrations at 6 hours, despite reduced cellular activity. Further modifications to the pyrrolidine ring were explored, revealing that fusion with a phenyl group (compound **52**) significantly improved enzymatic and cellular potency. However, **52** displayed limited oral bioavailability in rats (Figures 7C–E).

**FIGURE 8:**
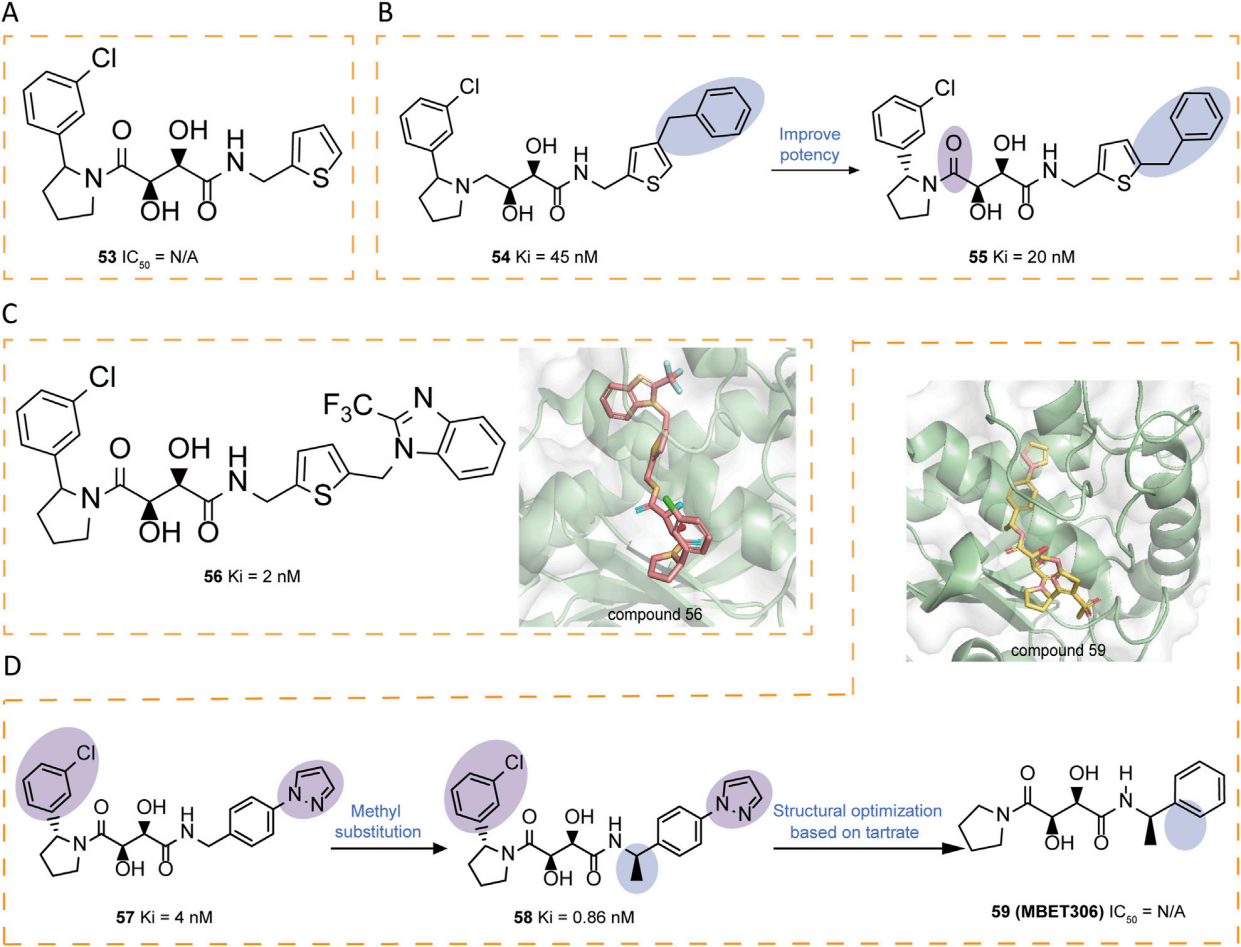
**(A)** Chemical structures of compounds 53. **(B)** Chemical structures and structure–activity relationships of compounds 54-55. **(C)** Chemical structures and crystal structure of catalytic domain of ADAM17 combined with compound 56 (PDB ID: 3LGP). **(D)** Chemical structures and structure–activity relationships of compounds 57-59 and crystal structure of catalytic domain of ADAM17 combined with compound 59 (PDB ID: 3O64).

Building upon tartrate diamide inhibitors like compound **53**, researchers identified 2-arylpyrrolidines as promising non-prime site moieties (Figure 8A). Introducing a benzyl group in compound **54** significantly enhanced potency compared to **53**, as it extended into the S3' region. Further exploration of the S3 region through phenyl ring modifications resulted in **55**, which served as a basis for additional optimizations. A key discovery was that replacing the 2,4-disubstituted thiophene ring in **55** with a 2,5-analog maintained comparable activity, prompting further investigations (Figure 8B). Crystallographic analysis of **56** confirmed the binding mode, providing structural evidence that informed subsequent design iterations (Figure 8C). A thorough investigation of prime site amide substitutions revealed that certain heterocyclic replacements and ortho-substituents, as exemplified in compound **57**, contributed to enhanced enzymatic potency and oral rat PK. A methyl substituent was introduced to mitigate possible metabolic oxidation of the benzylic moiety, yielding **58** (Ki = 0.86 nM) with improved stability (Li et al., 2010). Through molecular dynamics simulations, Banchelli et al. investigated the conformational dynamics of tartrate-based inhibitors, leading to the synthesis of compound **59** (MBET306) (Banchelli et al., 2013). In aqueous solution, this molecule predominantly adopts two distinct conformations: an extended form and a bent form, the latter stabilized by pseudo-stacking interactions between its terminal cyclic groups. Notably, both conformations show a ZBG arrangement which differs from previously reported ADAM17 co-crystal structures with tartrate-derived ligands (Figure 8D). Further analysis revealed that the monoanionic form of the bent conformation exhibits mild zinc affinity at physiological pH, triggering a conformational shift in the ZBG. This shift facilitates a predominantly tridentate coordination, where two hydroxyl groups and the LHS carboxyl group complex the zinc ion. These findings support a proposed two-step binding mechanism for tartrate-based ADAM17 inhibitors, in which an intermediate ADAM17-drug complex initially forms upon zinc docking, potentially influencing inhibitor design and optimization.

### 5.7 Other non-hydroxamate-based small-molecule inhibitors

**FIGURE 9:**
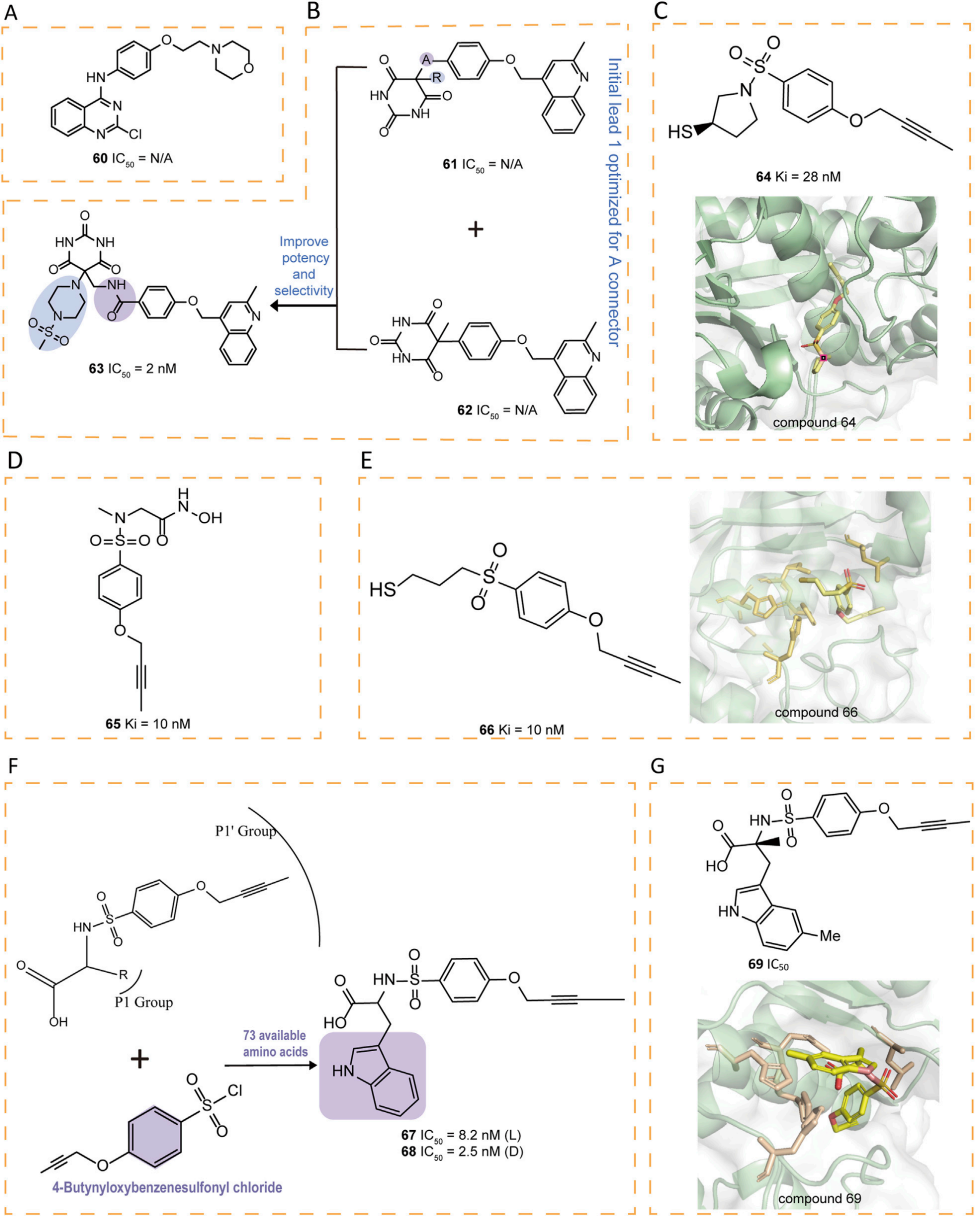
**(A)** Chemical structures of compound 60. **(B)** Chemical structures and structure‐activity relationships of compounds 61‐63. **(C)** Chemical structures and crystal structure of catalytic domain of ADAM17 combined with compound 64 (PDB ID: 2OI0). **(D)** Chemical structure of compound 65. **(E)** Chemical structures and crystal structure of catalytic domain of ADAM17 combined with compound 66 (PDB ID: 3B92). **(F)** Chemical structures and structure‐activity relationships of compounds 67‐68. **(G)** Chemical structures and crystal structure of catalytic domain of ADAM17 combined with compound 69 (PDB ID: 3G42)

Some quinazoline derivatives have been reported to possess potent anti-inflammatory activity as TNF-α inhibitors, with **60** (9c) being the first identified quinazoline derivative to inhibit ADAM17-mediated TNF-α production (Figure 9A) (Pu et al., 2015). Additionally, new inhibitors were discovered by incorporating a 4-(2-methyl-4-quinolinylmethoxy)phenyl group and an optimized selective P1' group, replacing the hydroxamic acid with a pyrimidine-2,4,6-trione (Duan et al., 2005). A detailed SAR investigation explored the influence of different linkers between the pyrimidine-trione and 4-(2-methylquinolin-4-ylmethoxy)phenyl groups in compound **61**, incorporating alkylene, alkenylene, ether, and amide modifications. Optimization of the initial lead compound **62** ultimately yielded **63** (Figure 9B) (Duan et al., 2007).

A series of thiol-containing aryl sulfonamides demonstrated potent enzymatic inhibition, with compound **64** exhibiting particularly remarkable 200-fold selectivity over MMP-2 and MMP-7 (Figure 9C) (Govinda Rao et al., 2007). Comparative analysis revealed that **64** exhibits marginally reduced potency compared to hydroxamic acid counterpart **65**, yet demonstrates enhanced selectivity profiles against MMP-2, -8, and -13. Structural investigations indicated that substituting the sulfonamide group with a sulfone group preserves both the bound conformation and aa the critical enzymatic interactions with the active site (Figure 9D). Notably, compound **66** emerged as a highly selective derivative, surpassing the MMPs selectivity of earlier analogs including **65** (Figure 9E). These findings collectively establish the structural tolerance for sulfone replacement within this molecular framework while maintaining pharmacological efficacy.

**FIGURE 10:**
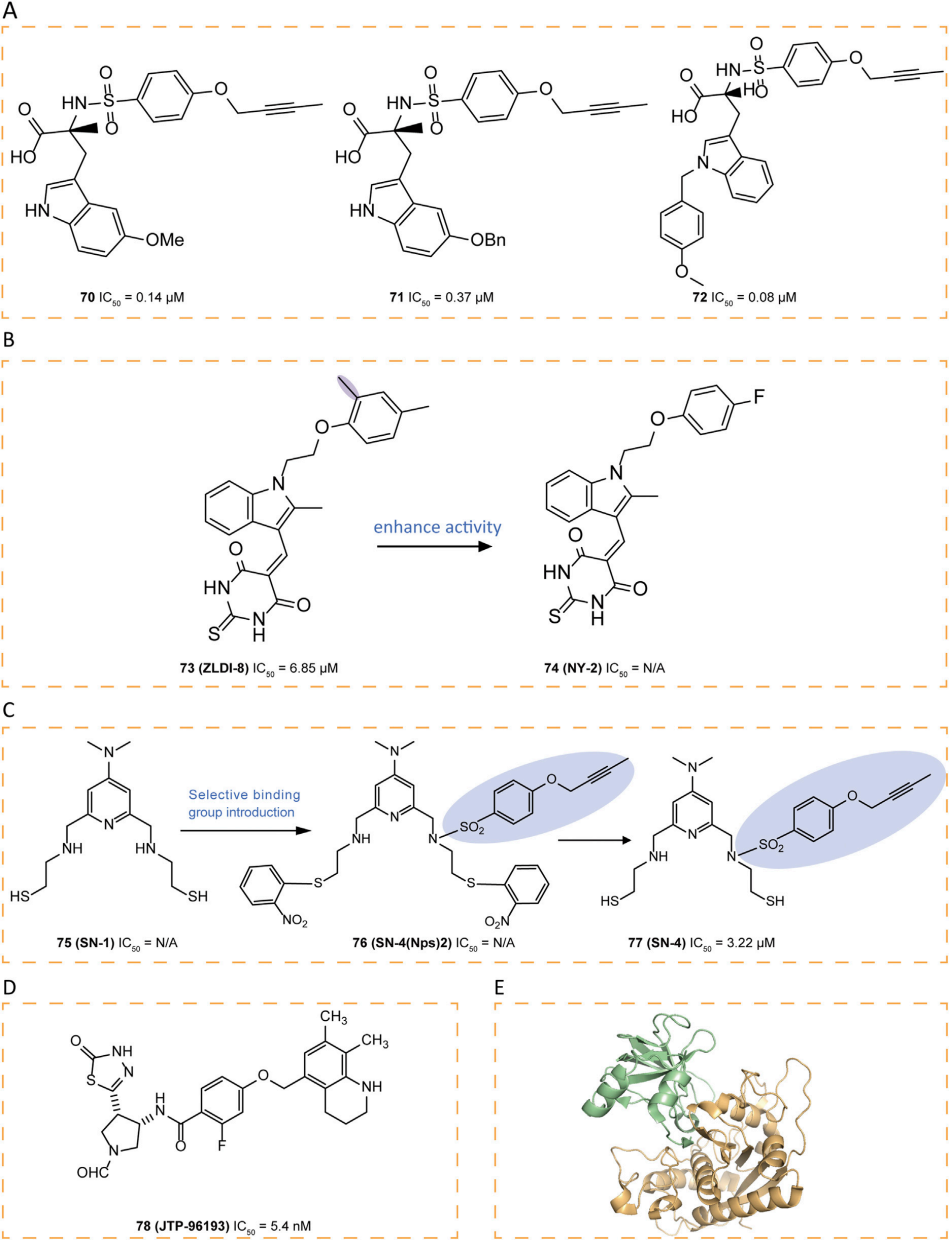
**(A)** Chemical structures of compounds 70-72. **(B)** Chemical structures and structure–activity relationships of compounds 73-74. **(C)** Chemical structures and structure–activity relationships of compounds 75-77. **(D)** Chemical structures of compound 78. **(E)** Crystal structure of the TACE-N-TIMP-3 complex (PDB ID: 3CKI).

Compounds **67–72** were tryptophan sulfonamide derivatives featuring a butynyloxy P1′ moiety. Initially, the reaction of 4-butynyloxybenzenesulfonyl chloride with 73 commercially available amino acids led to the identification of tryptophan sulfonamide derivatives **67** and **68** as the only compounds exhibiting single-digit micromolar inhibition (Figure 9F). Substitutions on the indole ring were explored to assess the SAR within this series. Four tryptophan carboxylate derivatives (**69–72**), which demonstrated promising activity, were chosen for selectivity profiling against MMPs (Figures 9G, 10A). The X-ray structure of **69** revealed that its carboxylate group coordinates the zinc ion at the active site, which is also ligated by His405, His409, and His415, while its 5-methyl indole moiety engages in hydrophobic interactions with Ala351 and Val353 and potentially forms a π interaction with His409, and its butynyloxy P1′ group extends into the narrow hydrophobic channel connecting the S1′ and S3′ pockets (Figure 9G). Among these derivatives, compound **72** exhibited the highest *in vitro* potency against isolated ADAM17 (Park et al., 2009).

Through computational virtual screening, thiodihydropyrimidinedione **73** (ZLDI-8) was discovered which can reverse paclitaxel resistance and inhibit the metastasis of hepatocellular carcinoma (Li et al., 2018; Lu et al., 2019; Lu et al., 2020). It can also sensitize hepatocellular carcinoma cells to sorafenib via the Notch1-integrin beta-talk (Xu et al., 2024). In pursuit of enhanced potency for **73**, a series of derivatives were synthesized, among which **74** (NY-2) emerged as the most potent candidate, demonstrating not only significantly improved pharmacokinetic properties with distinct advantages but also an absence of apparent *in vivo* toxicity (Zhang et al., 2023) (Figure 10B). These improvements are likely attributed to the electron-withdrawing effect of the fluorine atom introduced into the derivative, which reduces oxidative metabolism by enzymes involved in metabolic pathways.

Compound **75** (SN-1), a zinc-binding molecule contains two SH groups, can bind and modulate multiple zinc-containing proteins (Fujita et al., 1996). By introducing a 4-(but-2-yn-1-yloxy)benzenesulfonylamide group into **75**, which binds selectively to the S1′ pocket of ADAM17, selectivity was obtained and a derivative **77** (SN-4) was designed (Tateishi et al., 2021). Since thiols are prone to oxidation, forming disulfides upon exposure to air, a prodrug strategy was employed (Tateishi et al., 2021). The oxidative form of **75** produced by introducing two Nps groups as prodrugs was found to release **75** more effectively under intracellular reducing conditions than the intramolecular or intermolecular oxidative form of **75** (Radwan et al., 2019). As a result, **76** (SN-4 (Nps) 2) was designed and produced as a prodrug of **77**, which specifically inhibited ADAM17 with an IC_50_ of 3.22 μM (Figure 10C) (Tateishi et al., 2021). To confirm that this selectivity stemmed from the binding of its 4-(but-2-yn-1-yloxy)benzenesulfonyl amide group to the S1′ pocket, molecular docking studies were conducted. The result showed that the ADAM17 binding portion of **77** almost aligns with the structure of the co-crystallized compound **64**, fitting into the S1′ specificity pocket, threading through a narrow channel and extending into the S3′ subsite. This unique structural feature grants ADAM17 selectivity over other MMPs, where a conserved tyrosine obstructs this channel. Additionally, the sulfone linker stabilizes the complex through hydrogen bonding with Leu348 and Gly349, while the thiol group of **74** exhibits strong coordination with the active-site zinc ion, forming a stable tetrahedral complex with His405, His409, and His415. Simultaneously, the pyridinium nitrogen interacts with a water molecule (HOH578) through hydrogen bonding. Recent studies suggest that **77** not only enhances glucose uptake and lipid metabolism via AMPK activation—without increasing lactate production—but also inhibits TNF-α release from adipocytes, highlighting its potential as a novel anti-diabetic agent (Toma et al., 2024).

**78** (JTP-96193), a thiadiazolone derivative, demonstrated potent and selective inhibition of ADAM17 with an IC_50_ value of 5.4 nM, exhibiting over 1800-fold selectivity against other MMPs (Figure 10D). In obese and diabetic mouse models, **78** reduced the release of TNF-α from adipose tissue, prevented the development of diabetes and improved insulin resistance, which may be an adjunct to the treatment of type 2 diabetes associated with microvascular complications (Maekawa et al., 2019).

## 6 Monoclonal antibodies

Although ADAM17 is a well-established therapeutic target, small-molecule inhibitors designed to bind its active site have not succeeded in clinical trials, primarily due to challenges related to specificity, limited efficacy, and toxicity concerns. However, the emergence of monoclonal antibodies (mAbs) overcomes these issues.

Trad et al. employed hybridoma technology to develop three mAbs—**A300**, **A309**, and **A318**—targeting the extracellular domain of human ADAM17 (Trad et al., 2011). These antibodies, classified as IgG1 with κ light chains, exhibit distinct functional properties. **A300E**, which is rapidly internalized and exhibits an IC_50_ of approximately 0.7 mg/mL against ADAM17, facilitates the targeted delivery of conjugated toxins into cancer cells (Yamamoto et al., 2012; Trad et al., 2013). proceeded to develop a single-chain antibody as well as a bispecific anti-ADAM17 A300E-BiTE antibody that includes a CD3-binding segment (Yamamoto et al., 2012). **A9 (B8)**, which exhibits cross-reactivity with both human and mouse ADAM17, demonstrates EGFR-TKI-mediated antitumor effects in NSCLC cells, with IC_50_ values of 0.22 nM for human ADAM17 and 0.25 nM for mouse ADAM17 (Yang et al., 2019). It effectively inhibits ADAM17 substrate shedding and suppresses pancreatic ductal adenocarcinoma growth both *in vitro* and *in vivo* (Ye et al., 2017).

Research on ADAM17 inhibitors has primarily focused on inhibiting the active site; however, due to the high conservation of metalloprotease active sites, these approaches have failed to yield potent and specific inhibitors. To address this challenge, a “two-step” phage display technique was employed to develop a “cross-domain” human antibody, **D1 (A12)**, which represents a novel and selective ADAM17 antibody, providing an alternative to small-molecule metalloprotease inhibition (Tape et al., 2011). At a concentration of just 4.7 nM, **D1 (A12)** achieved 50% inhibition of TNF-α shedding and demonstrated anti-ovarian cancer activity (Richards et al., 2012). Furthermore, it suppresses the progression of squamous cell carcinoma by reducing delayed hormone-induced HERS trans-activation and holds therapeutic potential for overcoming EGFR TKI resistance (Huang et al., 2014).

Another monoclonal antibody, **MEDI3622,** effectively inhibits EGFR activity, demonstrating potent efficacy with an IC_50_ value of 39 pmol/L against ADAM17. Furthermore, **MEDI3622** enhances the immune response by blocking the shedding of CD16A, which increases antibody binding on natural killer (NK) cells and promotes the release of tumor cells bound to IFN-γ (Rios-Doria et al., 2015). **D8P1C1**, an affinity-matured fully human mAb, binds the MD domain of ADAM17 and inhibits its enzymatic activity (Saha et al., 2022). **C12 mAb**, which targets the cysteine-rich domain of ADAM17, demonstrates a moderate anti-proliferative effect across a range of cancer cell lines. By inhibiting the cleavage of EGFR ligands, the **C12 mAb** effectively suppresses EGFR phosphorylation in these cancer cell lines, highlighting its potential as a therapeutic agent (Saha et al., 2024).

## 7 Endogenous protein inhibitors

Tissue inhibitors of metalloproteinases (TIMPs) are endogenous inhibitors of MPPs and play a crucial role in regulating ECM turnover, tissue remodeling, and cellular processes (Brew and Nagase, 2010). Among them, TIMP3 is the only protein that binds to the ECM and contains the amino acid sequence required to inhibit ADAM17 (Lee et al., 2005). Studies have shown that TIMP-3-mediated suppression of ADAM17 regulates TNF-α levels *in vivo* and prevents spontaneous inflammation (Mohammed et al., 2004; Murthy et al., 2012b; Murthy et al., 2010). Structural analysis indicates that TIMP-3 interacts with ADAM17 through a mechanism similar to its binding with MMPs (Figure 10E) (Carreca et al., 2020). Specifically, the Phe34 side chain, positioned at the tip of the short sA-sB loop, extends into a distinct hydrophobic groove on the ADAM17 surface, while two adjacent leucine residues from the sC-connector and sE-sF loops form a tightly packed interface, enhancing binding stability. The combination of these functional epitopes and structural flexibility makes TIMP-3 a highly effective ADAM17 inhibitor. Understanding this interaction may facilitate the design of more potent TIMP-based ADAM17 inhibitors (Wisniewska et al., 2008).

## 8 Conclusion

ADAM17 is a crucial transmembrane protease involved in various physiological and pathological processes, including inflammatory responses, cell proliferation, and cancer progression. As a result, ADAM17 inhibitors are considered promising therapeutic targets for multiple diseases and have shown significant potential in clinical research. This paper reviews the latest advancements in ADAM17 inhibitors, including different types of small-molecule inhibitors. However, major challenges associated with current ADAM17 inhibitors include low specificity, significant off-target effects, and potential side effects, which have limited their further clinical applications. Additionally, many existing ADAM17 inhibitors suffer from poor bioavailability and limited cell membrane permeability, indicating the need to optimize their physicochemical properties to enhance efficacy and safety.

Therefore, developing novel small-molecule ADAM17 inhibitors is of paramount importance. There are two primary strategies for ADAM17 inhibition: one involves directly targeting the catalytic domain of ADAM17 to improve selectivity and efficacy, such as small-molecule inhibitors; the other involves using antibodies or biologics to selectively block ADAM17’s substrate-binding site. Additionally, since iRhom2 is a key regulator of ADAM17 activity, targeting iRhom2 has emerged as a promising alternative strategy for ADAM17 inhibition (Maciag et al., 2024). Targeted protein degradation (TPD) techniques, particularly proteolysis-targeting chimeras (PROTACs) and molecular glue degraders (MGDs), have introduced innovative strategies in drug discovery (Lv et al., 2025). As an emerging protein degradation approach, PROTAC technology can selectively degrade ADAM17 through the ubiquitin-proteasome pathway, offering significant potential in overcoming drug resistance and targeting “undruggable” proteins. Although no PROTAC-based ADAM17 inhibitors have been reported yet, this strategy could pave the way for the future development of ADAM17-targeted therapeutics.

Given the widespread expression of ADAM17 in various tissues and cell types, achieving a balance between efficacy and safety is paramount in the development of ADAM17 inhibitors. Given the central role of ADAM17 in numerous diseases and the advances in current research, there is an urgent need to discover novel, potent inhibitors with minimized side effects. As biotechnology and pharmaceutical chemistry advance, it will be essential to conduct rigorous clinical trials to thoroughly evaluate efficacy, safety, and optimal drug delivery strategies, including dosing regimens, to improve therapeutic outcomes. By leveraging advanced molecular pharmacology and high-throughput drug screening technologies, scientific breakthroughs in ADAM17 research can be more effectively translated into clinical applications. As a result, we are confident that ADAM17 inhibitors represent a promising therapeutic avenue and merit further investigation.

## 9 Future perspective

As the understanding of ADAM17’s mechanisms and its interactions with inhibitors deepens, novel therapeutic strategies are expected to emerge, particularly those focusing on selective inhibition of ADAM17 isoforms to enhance therapeutic efficacy while reducing side effects. Targeting specific structural domains of ADAM17, such as the catalytic or regulatory domains, could improve inhibitor selectivity and minimize off-target effects. The application of allosteric modulators interacting with unique ADAM17 conformations may further enable the design of more precise inhibitors with improved safety profiles.

Addressing the side effects of ADAM17 inhibition, such as immune suppression or tissue damage, requires a more refined approach, including optimizing administration routes and developing inhibitors with improved pharmacokinetics and bioavailability. The combination of ADAM17 inhibitors with anti-inflammatory agents or immunomodulators may offer synergistic effects, enhancing therapeutic outcomes while allowing for lower dosages, thereby reducing potential adverse effects.

Additionally, dual-target and combination therapy approaches may offer promising avenues for future development. The simultaneous inhibition of ADAM17 and other key proteases involved in disease progression could result in synergistic therapeutic effects. Furthermore, emerging technologies such as PROTAC-mediated ADAM17 degradation represent an innovative strategy to overcome drug resistance and target previously undruggable aspects of ADAM17 function.

With advancements in biotechnology and medicinal chemistry, future research will focus on designing next-generation ADAM17 inhibitors with enhanced specificity, reduced toxicity, and optimized therapeutic potential. The ongoing exploration of novel ADAM17-targeting strategies highlights its promising role in disease treatment, making it a key area of interest in drug discovery and clinical research.
